# “Planeterranea”: An attempt to broaden the beneficial effects of the Mediterranean diet worldwide

**DOI:** 10.3389/fnut.2022.973757

**Published:** 2022-09-02

**Authors:** Claudia Vetrani, Prisco Piscitelli, Giovanna Muscogiuri, Luigi Barrea, Daniela Laudisio, Chiara Graziadio, Francesca Marino, Annamaria Colao

**Affiliations:** ^1^Department of Clinical Medicine and Surgery, Endocrinology Unit, University of Naples “Federico II”, Naples, Italy; ^2^Centro Italiano per la Cura e il Benessere del Paziente con Obesità (C.I.B.O), University of Naples “Federico II”, Naples, Italy; ^3^UNESCO Chair “Education for Health and Sustainable Development, ” University of Naples “Federico II”, Naples, Italy; ^4^Dipartimento di Scienze Umanistiche, Università Telematica Pegaso, Napoli, Italy

**Keywords:** Mediterranean diet, health, sustainability, local foods, nutritional properties, bioactive compounds, nutritional pyramid

## Abstract

Non-communicable diseases (NCDs) lead to a dramatic burden on morbidity and mortality worldwide. Diet is a modifiable risk factor for NCDs, with Mediterranean Diet (MD) being one of the most effective dietary strategies to reduce diabetes, cardiovascular diseases, and cancer. Nevertheless, MD transferability to non-Mediterranean is challenging and requires a shared path between the scientific community and stakeholders. Therefore, the UNESCO Chair on Health Education and Sustainable Development is fostering a research project—“Planeterranea”—aiming to identify a healthy dietary pattern based on food products available in the different areas of the world with the nutritional properties of MD. This review aimed to collect information about eating habits and native crops in 5 macro-areas (North America, Latin America, Africa, Asia, and Australia). The information was used to develop specific “nutritional pyramids” based on the foods available in the macro-areas presenting the same nutritional properties and health benefits of MD.

## Introduction

Non-communicable diseases (NCDs) account for more than 70% of global mortality (1). A recent report from the Global Burden Disease Study reported that almost 400 million people are suffering from diabetes (2) and the presence of diabetes is associated with increased mortality from infections, cardiovascular disease (CVD), and cancer (2, 3). Other to diabetes, high systolic blood pressure and high low-density lipoprotein cholesterol levels have been appointed as major contributors to the onset of CVD (54.6 and 46.6%, respectively) (4). Noteworthy, a 26.3% increase in new cases of cancer (mainly breast and gastrointestinal cancers) has been reported recently (5).

Given the epidemic of NCDs with increasing trends in high and middle-income countries and in adolescents/young adults (6, 7), the World Health Organization demanded for effective strategies to treat and prevent NCDs. In addition to lifestyle factors such as physical inactivity, smoking, alcohol intake, also diet has been established as highly modifiable risk factors for NCDs. Indeed, more than 9.1 million premature deaths from CVDs worldwide are attributable to dietary risks, regardless of age, sex, and sociodemographic development of the native country (8). As for cancer (breast and gastrointestinal cancers), nutritional inadequacy, and excess body weight (overweight and obesity) lead to a significant increase in cancer incidence during the last decades (9, 10).

Different dietary approaches have been proposed for the prevention and treatment of NCDs. Among this, Mediterranean diet (MD) have been associated with body weight control (11, 12) and reduced risk for chronic diseases, including CVD (13, 14), type 2 diabetes (T2D) (15), and some cancers (16–20).

Moreover, MD has shown a protective role also for immune-related diseases such as atopy and asthma (21, 22).

These beneficial effects are related to the nutritional composition of MD that can be obtained through the combination of some foods with a specific frequency of consumption during the week (23): (a) regular consumption of plant-based foods (fruits, vegetables, wholegrain, legumes, and nuts), and extra-virgin olive oil as the primary source of fat; (b) moderate amount of animal protein and fat, with fish and low-fat dairies as the preferred sources, respectively; (c) limited intake of sweets and processed foods. More in detail, most of MD energy intake is provided by non-refined carbohydrate (55–60%), 30–35% from fat, and ~15% from protein (24). Carbohydrate is provided by low-glycemic index foods (i.e., wholegrain-based products and legumes) while sugar intake is <10% by limiting the consumption of sweets and sugar-sweetened beverages. Fat is mainly represented by monounsaturated fatty acids (MUFA, 19%), followed by saturated fatty acids (SFA, 9%) and polyunsaturated fatty acids (PUFA, 5%), and cholesterol is 300 mg/day (24). According to MD, plant protein should be preferred, while animal protein (fish, lean cuts of meat, eggs, and dairies) should be used as alternative options during the week (25). MD can provide relevant amounts of vitamins, minerals, and other phytochemicals (23). Over the recommendations for the frequency of food consumption, MD relies on cultural, social, and lifestyle features. In brief, a moderate intake of wine and other fermented beverages (women: one glass/day; men two glasses/day) is advised while respecting religious and social beliefs. A daily intake of 1.5–2 l of water (6–8 glasses) is recommended for proper hydration. Regular physical activity (i.e., sports, fitness, or leisure activities outdoors) should complement diet in order to sustain adequate body weight and other health benefits. Finally, seasonality of foods, biodiversity preservation, conviviality, and socialization are advocated to preserve MD cultural heritage (23).

Given the beneficial effects of MD, several efforts have been made to transfer this dietary pattern to non-Mediterranean populations for the prevention of NCDs. Nevertheless, several barriers toward adherence to MD in non-Mediterranean countries exist. Firstly, it is known that changing individual dietary habits is challenging, as it requires long-lasting modifications in behavior (26). Furthermore, there are practical, cultural, and economic factors that affect dietary changes (27). In brief, the main issues related with poor compliance to MD are affordability (high cost for transportation and commercialization), lack of knowledge (health benefits, cooking methods, recipes), and cultural differences (traditions, native crops, and environment protection *vs*. globalization).

On the other hand, in recent years there is a growing recognition that “healthy nutrition” should be integrated with food systems, i.e., “all the factors (environment, people, inputs, processes, infrastructures, institutions, etc.) and activities that relate to the production, processing, distribution, preparation and consumption of food, and the outcomes of these activities” (28). This would lead to a more sustainable diet, with relevant benefits on the health of people and the planet (29).

In line with this, the UNESCO Chair of “Health Education and Sustainable Development” of the University of Naples “Federico II” has focused part of its activities on the implementation of MD-based dietary patterns as a pivotal approach to prevent and manage NCDs. This specific task of the Chair represents a response to the call of the World Health Organization to reduce the pandemic of NCD and their risk factors (30). Indeed, the long-term objective of the UNESCO Chair of “Health Education and Sustainable Development” is to enhance the health status of populations by facilitating a “Knowledge Transfer Exchange” about the effects on human health of major cultural, nutritional, and environmental factors (30).

Against this complex background, the aim of the present review was to collect the available evidence on native crops and dietary habits worldwide, grouping the countries into 5 macro-areas (North America, Latin America, Africa, Asia, and Australia). Then, the information retrieved was used to propose feasible alternatives with similar composition of foods characterizing MD. Finally, we used all the information to develop specific “nutritional pyramids” based on the foods available in the 5 macro-areas presenting the same nutritional properties and health benefits (as well as environmental-friendly production processes) observed for MD.

## Methods

We used the UNESCO network to retrieve information about dietary habits of different countries. In brief, we interviewed native people from the 5 macro-areas to collect information about local crops, eating habits, traditional recipes, etc. In addition, we performed complementary research by selecting national and international websites providing information about crop production and eating habits (i.e., National Department of Agriculture, USDA, Google Scholar, SciELO—Scientific Electronic Library Online, etc). Then, a 20-years literature search for this narrative review was conducted until December 2021 by searching PubMed database for articles published in the English language. Each specific food identified in the preliminary search was used as keyword “AND” (Boolean operator) combined with “health OR plasma glucose OR glucose metabolism OR plasma lipid OR lipid metabolism OR plasma insulin OR insulin resistance OR inflammation OR oxidative stress OR type 2 diabetes OR cardiovascular disease OR cancer OR risk factors OR effects OR composition OR intake OR consumption.”

Overall, our search retrieved a total of 860 studies suitable for our review. The citation pool of publications was further supplemented by analyzing the reference list of the selected articles.

Articles considered in this narrative review were (a) meta-analyses of prospective studies or randomized clinical trials; (b) observational studies and clinical trials not included in the meta-analyses that added significant information; (c) literature reviews providing the nutritional composition of the specific foods identified. Only articles published in journals in the highest impact factor quartile in the “Public Health,” “Endocrinology, Diabetes and Metabolism” or “Nutrition and Dietetics” areas were included. Moreover, we excluded notes, book chapters, letters, editorials, conference papers, articles published in languages other than English and those not specifically related to each issue of interest.

## Results

### North America

North America has been appointed as the cradle of the so-called Western diet, characterized by high intakes of refined grains, red and processed meat, pizza and fast food, potato, sweets, sugar, and high-energy beverages. It translates in increased amount of SFA (12%), refined carbohydrates (21% refined grains, fruit juice, and potatoes), and added sugars (14.4%) (31). These dietary features have been associated with poor diet quality and are a primary cause for chronic diseases and mortality in USA (32). Therefore, new approaches for promoting healthy dietary choices are advocated. Although massive changes are required to increase diet quality, the introduction of some local products could represent a first step to improve dietary habits.

Canola oil is a vegetable oil derived from a variety of rapeseed *Brassica napus* and *Brassica rapa* that originated in Canada. It contains MUFA (54% oleic acid), PUFA (21% linoleic acid and 11% a-linolenic acid), and high concentrations of phytosterols (769 mg/100 mg canola oil) (33, 34).

Mounting evidence supports the hypocholesterolaemic effect of canola oil. Indeed, two meta-analyses of randomized controlled trials have shown that replacing SFA or sunflower oils with canola oil significantly reduces total and LDL-cholesterol concentrations (35, 36). Moreover, a non-linear dose-response curve suggested that replacing ~15% of total caloric intake with canola oil might provide the greatest benefits on lipid profile (36). Besides, a recent meta-analysis demonstrated that canola oil can positively affect body weight, with no major changes of other anthropometric parameters (37). The effect is greater in studies in women, patients with T2D, and when canola oil is compared to saturated fatty acids.

Pecans *(Carya illinoensis (Wangenh.) K.Koch)* are considered as the traditional tree nut in North America. They are an excellent source of MUFA, having 12 g/standard serving (28 g), and γ-tocopherol (24.4 mg/100 g) (38). Moreover, they present the highest total flavonoid content among nuts (34 mg/100 g), consisting mostly of flavan-3-ols and anthocyanins (39).

Human studies with pecans supplementation have addressed mainly to lipid profile. In a 4-week trial, 23 middle aged healthy volunteers were randomized to pecan supplementation (72 g/day) in addition to the habitual diet or habitual diet alone. At the end of the study, participant following pecan supplementation presented a significant reduction of total and LDL-cholesterol, and increased HDL-cholesterol concentrations, with no changes of body weight. In addition, a significant reduction of triglyceride, lipoprotein (a), and apolipoprotein B concentrations was detected (40).

Conversely, a 12-week healthy diet enriched with pecans (30 g/day) did not improve lipid profile in patients with stable coronary artery disease suggesting that the threshold of pecan doses might be higher than the recommended portion for other nuts (41).

Over the hypolipidemic effect, a 4 week incorporation of 42 g/day of pecan in a typical American diet has shown to improve glucose metabolism in middle-aged volunteers with central adiposity. In particular, the pecan-enriched diet significantly reduced fasting insulin and insulin resistance (evaluated as homeostatic model assessment, HOMA), while improving b-cell function (assessed as HOMA-β) (42).

Okra (*Abelmoschus Esculentus*) is a popular vegetable crop cultivated throughout the world mostly in tropical and subtropical regions. It is the main ingredient of the “Gumbo,” a popular American dish, particularly in Louisiana.

Okra is rich in fibers (8.16 g/100 g), both insoluble (4.73 g/100 g) and soluble (3.43 g/100 g), and it has been suggested that 100 g of okra could cover a 33% of the recommended daily fiber intake. It has 2% protein, 7% carbohydrates and traces of fat (43).

To the best of our knowledge, no clinical studies with okra supplementation are available so far.

However, several *in vitro* and animal studies suggested potential antidiabetic and lipid-lowering effects by virtue of the high fiber content (44, 45).

Pinto Beans *(Phaseolus vulgaris L.)* is the major dry beans in terms of production (31%) and consumption (20%) in North America (46). They provide 8–10% of the daily recommended amount of protein *per* 100 g serving and have a great fiber content (16 g/100 g) (47).

A randomized controlled crossover trial with a 3 × 3 block design tested the effect of Pinto beans incorporation into the diet for 8 weeks as compared to black-eyed peas and carrot used as control (1/2 cup/day for all tested foods). Sixteen mildly insulin resistant adults completed the trial, and an 8% reduction of LDL-cholesterol was detected after the consumption of Pinto beans (48). Interestingly, it has been reported that 1% reduction in LDL-cholesterol reduces risk for coronary heart disease (CHD) by ~1% (49). Therefore, the reduction observed after Pinto beans consumption may translate in an 8% reduction of CHD risk. Similar results were obtained in a longer-term study (12 weeks) where the same amount of Pinto beans was consumed by individual with pre-Metabolic Syndrome (MS) (increased central adiposity and one clinical feature considered in the diagnosis of MS) (50). In this study, complementary experiments in an *in vitro* colonic fermentation model showed a significant increase of propionate with Pinto beans incubation. Therefore, it can be hypothesized that the LDL-cholesterol lowering effects of Pinto beans may be linked to the microbial products (mainly propionate) derived from fiber fermentation.

As reported above, massive changes are required to improve the overall quality of the actual North American diet. The Harvard School of Public Health and other American Institution, both have proposed practical guidelines to enhance the transferability of the MD in real-life settings (32). However, to increase the feasibility of these dietary changes, the incorporation of local foods rich in fiber, phytochemical and other bioactive compounds may represent a complementary strategy. Therefore, over the main principles of the MD for animal protein-sources and sweets, the following changes are proposed:

- use canola oil as the main daily fat source (the amount should be according to individual energy needs)
- increase the intake of vegetables (at least 2 servings/day) and legumes (at least 3 serving/week), preferring local varieties (i.e., okra and pinto beans)
- use nuts for snacking, in particular pecans (42 g/day)

**Figure 1:**
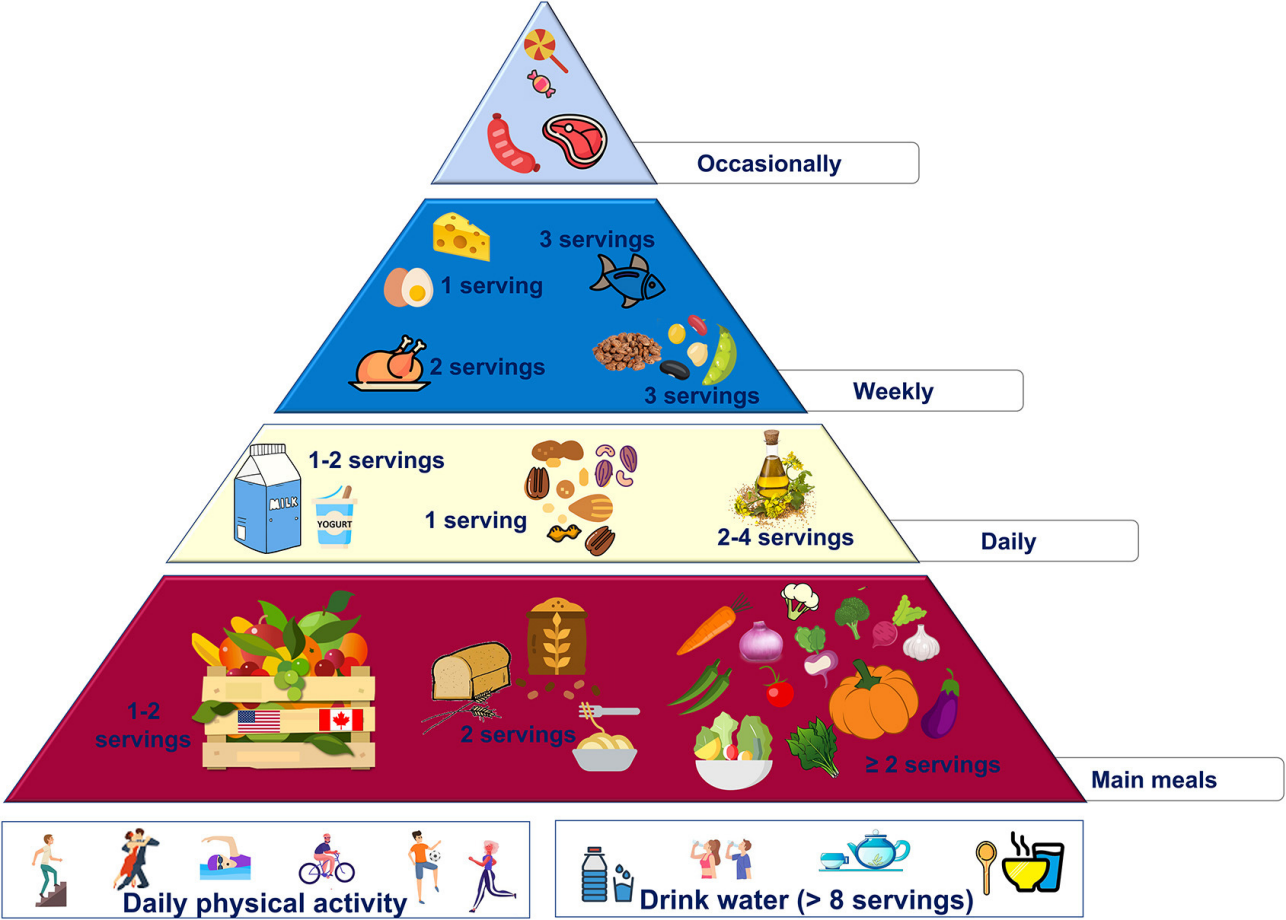
Proposed nutritional pyramid for North America.

These changes are summarized in the new food pyramid for North America (Figure 1).

### Latin America

The traditional Latin American diet is mainly characterized by the consumption of plant-based foods, i.e., fruit, vegetables, legumes, nuts, and seeds. Starchy foods habitually consumed are corn, potatoes, and rice. Red meat is the main protein source while small portion of animal protein are provided by poultry, eggs, fish, seafood, and dairy. Therefore, the overall daily macronutrients distribution is balanced, with 54% carbohydrate, 30% fat, and 16% protein intakes (51).

Nevertheless, many nutritional surveys have reported that Latin Americans, particularly those living in urban areas or in low-socioeconomic conditions, presented low diet quality and variety. More in details, over 25% of total energy intake was provided by high glycemic index (i.e., white rice and potato), and sugar and fat-rich ultra-processed foods (i.e., ready-to-eat foods, sugar-sweetened beverages, pastries, chips, and candies). In addition, the consumption of foods rich in fiber, micronutrients, and phytochemicals (i.e., whole grains, fruits, vegetables, beans, and nuts) represented only 17.7% of total energy intake (51, 52).

These nutritional changes have unfavorable effects on blood glucose homeostasis and lipid profile, and possibly explain the high prevalence of obesity and cardiometabolic diseases in Latin American populations (53).

Therefore, preferring the consumption of low-glycemic index starchy foods and foodstuffs with hypolipidemic and antioxidant properties is an essential shift to accomplish. In addition, promoting the consumption of diverse local foods may improve the overall diet quality in these populations.

Quinoa (*Chenopodium quinoa Willd*.) is a pseudocereal native to the Andean region. Due to its small size its botanical parts can not be separated during the milling process. Therefore, quinoa is considered as a whole grain. It has a higher total protein content (12.9–16.5%) compared to other cereals (but equal to wheat) and provides all essential amino acids (54). Fat content ranged 5–9%, which is higher than other cereals, and mainly consists in oleic acids (19.7–29.5%), linoleic (49.0–56.4%), and α-linolenic (8.7–11.7%) acids. It exhibits a low glycemic index (ranging 35–53) by virtue of its small granules (particles <2 μm in diameter), and 10% total dietary fibre, mainly soluble types (55). As compared to other cereals, 100 g of quinoa also contains higher amount of calcium (149 mg), iron (13.2 mg), and potassium (927 mg) (56).

It is also a good source of vitamins and other phytochemicals, such as polyphenols (251.5 mg/g ferulic acid, 0.8 mg/g *p*-coumaric acid, and 6.31 mg/g caffeic acid) and phytosterols (118 mg/100 g) (57, 58). Notably, it was reported that one serving of quinoa consumption (40 g raw) meets an important part of daily recommendations for essential nutrients (i.e., zinc, folic acid, magnesium, copper, and phosphorus) (59).

As for the health benefits of quinoa consumption, few human studies have been carried out so far. Recently, a meta-analysis of randomized controlled trials has reported that quinoa supplementation (20–65 g/day) may represent a useful strategy to manage cardiovascular risk factors in general adult population (60). Indeed, the addition of quinoa to the habitual diet significantly affected anthropometric parameters (body weight, waist circumference, and fat mass) as well as fasting insulin and blood lipid concentrations in individuals with overweight obesity or pre-diabetes. The mechanisms behind these effects are still unclear (61, 62). Nevertheless, it can be speculated that fiber can be fermented by colonic microbiota with the production of short chain fatty acids (SCFAs), as occur for other wholegrains. SCFAs has been proven to modulate appetite feeling, and to improve insulin sensitivity and lipid metabolism (63).

Plátanos (*Musa sp*. also known as “green bananas”) are starchy foods used in Caribbean and Northern Latin American cooking. They contain elevated total carbohydrates amount (73.5%), but they are mainly represented by indigestible carbohydrates, i.e., resistant starch and other fibers (cellulose, hemicelluloses, and lignin) (64). Resistant starch has shown to reduce postprandial glucose response in individuals with overweight/obesity (65) as well as in patients with T2D (66). In addition, it promotes colonic fermentation and improve the gut microbiota composition, possibly influencing insulin sensitivity and other cardiovascular risk factors (67, 68).

Human studies with platano and its derived products (starch) provided promising results. A short-term trial (45-day) in 25 postmenopausal women with MS tested the effects of plátanos addition (20 g/day) to the habitual diet. At the end of the study, a significant improvement of fasting glucose concentrations and systolic blood pressure were observed, with no changes in body weight or composition. These findings have been related to the low glycemic index of platano (15.3) assessed during the study, although the possible effects of other bioactive compounds can not be excluded (i.e., polyphenols, vitamins, and minerals) (69).

Platano starch supplementation was used in trials carried out in patients with T2D. In 4-week crossover trial 28 middle aged patients with T2D underwent a nutritional intervention either with starch (24 g/day) or soy milk (24 ml/day) dissolved in 240 mL of water. As compared to soy milk, starch supplementation induced a greater body weight loss, with no major changes in blood glucose control and lipid concentrations. Moreover, a reduction of insulin resistance (measured as HOMA-IR) was observed after starch supplementation, driven by decreased fasting insulin levels (70). In a more recent trial with longer duration (6 months), the supplementation with 4.5 g/day was tested in a group of patients with prediabetes or T2D (*n* = 61 starch vs n = 52 control). Starch significantly affected anthropometric parameters (body weight, waist, and hip circumferences, body fat) and blood pressure compared to control (71). Moreover, a significant improvement of fasting glucose and glycated hemoglobin (HbA1c) concentrations was observed after starch supplementation. These effects were obtained with no changes of the overall dietary composition, thus suggesting an independent role of starch in the improvement of metabolic control and body composition.

Avocado (*Persea americana*) is one of the main fat sources in Latin American diet and it might be preferred to olive oil due its lower cost, particularly among low-income individuals.

Avocados are rich in monounsaturated fatty acids (9.8/100 g), vitamin E (1.97 mg/100 g), and polyphenols (almost 200 mg/100 g), which can contribute to reduction of CVD risk (72). It has been reported that one avocado (136 g) has nutrient and phytochemical profiles similar to 40 g of nuts (72) and presents the same MUFA amount contained in almost 18 ml of olive oil (73).

The main health benefits of avocado consumption are related to the improvement of blood lipid concentrations. Indeed, several meta-analyses (74–76) have reported a moderate to large effect on LDL cholesterol (−3.54 mg/dl; 95% CI: −9.66, 2.58 mg/dl) even great heterogeneity was observed among studies. Moreover, avocado consumption (1 to 3 avocado/day) has shown to significantly increase HDL-cholesterol concentrations (3.90 mg/dl; 95% CI: 0.44, 7.36 mg/dl) (75). In addition, the consumption of one avocado/day (almost 136 g) has shown to improve oxidative stress (77) and cognitive function (78) in individuals with overweight/obesity by virtue of its bioactive compounds with antioxidant properties, in particular polyphenols (almost 200 mg), lutein and zeaxanthin (370 μg) (72).

Açaí berries (*Euterpe oleracea Mart*.) are the fruit of a typical palm tree found in the floodplains along the Amazon River estuary. They can be green (white açaí) or purple (dark açaí) according to the variety with no difference in nutritional composition. They are good sources of fat (32–48 g/100 g, mainly omega 6 and 9), fiber (44 g/100 g) polyphenols (424.9 mg/100 g), particularly anthocyanins (cyanidin 3-glucoside, cyanidin 3-rutinoside, and peonidin-3-rutinoside), proanthocyanidins, phenolic acids (gallic, protocatechuic, p-coumaric, ellagic, vanillic, and syringic acids), stilben (resveratrol), and other flavonoids (epicatechin, quercetin, catechin, velutin, homoorientin, orientim, isovitexin, and taxifolin deoxyhexosein) (79).

Few human trials evaluating the effects of *Aça*í berries on health are available. In a short-term trial (4 weeks), 10 overweight volunteers were asked to consume 200 g/day of açai pulp. At the end of the study, an improvement of fasting glucose, total and LDL-cholesterol concentrations was observed (80). The same amount of açai pulp was used in a study focusing on the potential antioxidant activity of açai berries in 35 young women (81). After 4-weeek, açai pulp increased the activity of antioxidant enzymes, total antioxidant capacity, and reduced the production of reactive oxygen species and protein carbonyl concentrations. In a more recent randomized, double-blind, placebo-controlled clinical trial, 69 overweight dyslipidemic patients were assigned to a hypocaloric diet + 200 g/day of açai pulp or hypocaloric diet alone. The hypocaloric diet was designed to achieve a 6 kg-weight loss with a balance macronutrient distribution (protein 15%, 45–60% carbohydrate, and 25–30% fat). After 8 weeks, in the group consuming acai pulp, a significant improvement of oxidative stress (measured as plasma 8-isoprostane concentrations) and inflammation (evaluated as IL-6 and IFN-γ) was observed (82).

**Figure 2:**
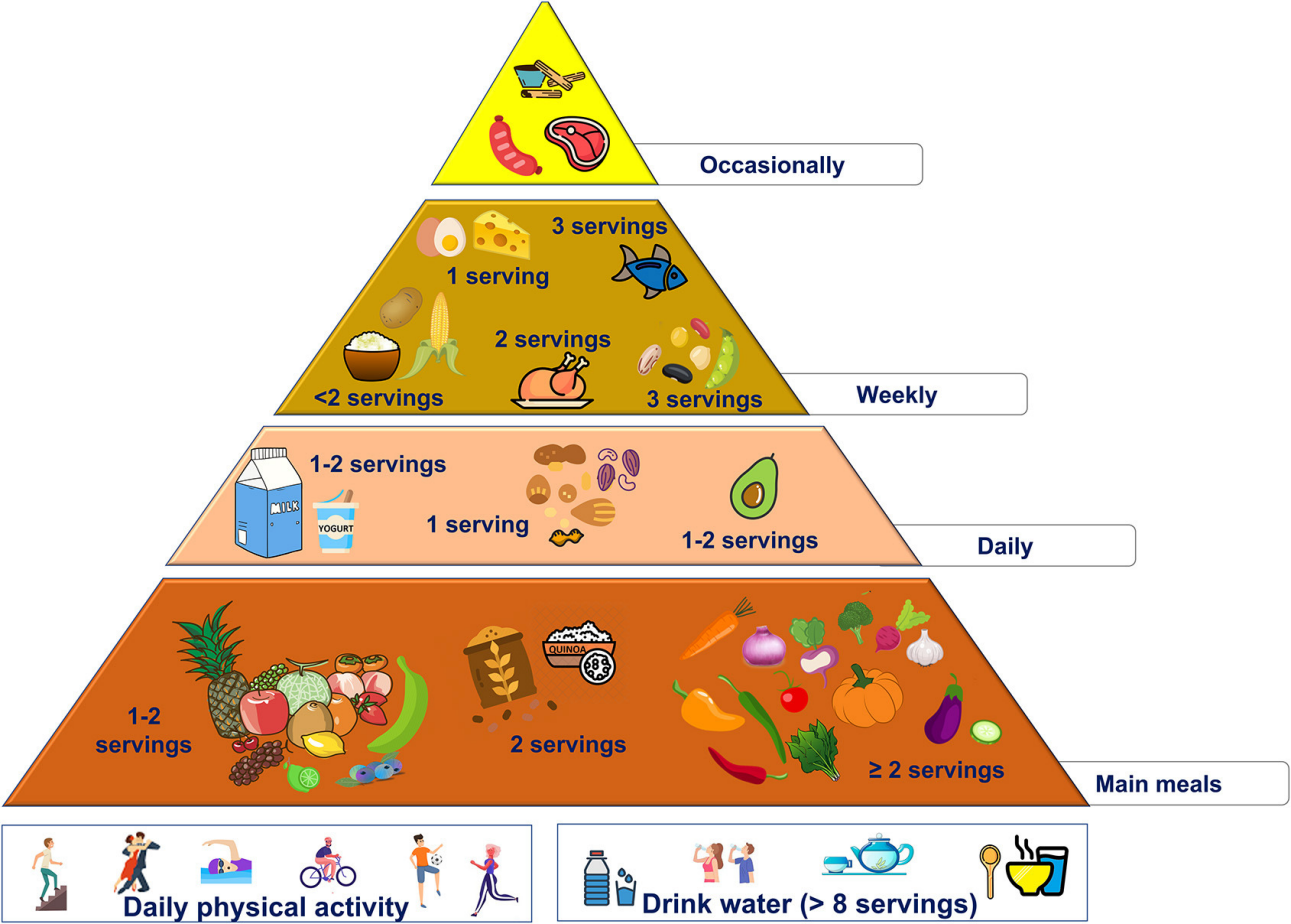
Proposed nutritional pyramid for Latin America.

As reported above, the traditional Latin American diet is a plant-based dietary pattern and provides adequate amount of the main macro- and micronutrients. However, globalization has induced a shift toward Western dietary habits. In addition, some traditional foodstuffs should be limited and substituted with other having more beneficial characteristics. Therefore, over the main principles of the MD for animal protein-sources and sweets, the following changes are proposed:

- 2 servings/day of starchy foods with low glycemic index (quinoa, plátanos, and wholegrain)
- no more than 2 servings/week of starchy foods with high glycemic index (rice, corn, and potato)
- use avocado as the main daily fat source (the amount should be according to individual energy needs)
- increase the intake of vegetables (at least 2 servings/day) and fruits (1 or 2 servings/day), preferring fruit with high antioxidant activities (like açai and other berries).
- These changes are summarized in the new food pyramid for Latin America (Figure 2).

### Africa

Although malnutrition and food shortages are still prevalent in this continent, many African countries underwent the “nutrition transition” during the last years. Indeed, very recently the profound changes that have taken place in Africa have been reported (83). Overall, these dietary changes consist of a shift from the consumption of natural foods with marginal handling—mainly fruit, vegetables, and legumes- to highly processed foods (refined carbohydrates, SFA and trans fats, added sugars, salt, and food additives). Notably, this phenomenon occurred also in African Mediterranean countries (Egypt, Libya, Tunisia, Algeria, and Morocco) (84). Unfortunately, to the best of our knowledge, no clear information on the actual nutritional habits of African populations is available so far. This lack of information is mainly due to methodological limitations in the assessment of dietary intake as reported in previous studies (85).

Nevertheless, as for other continents, it could be hypothesized that promoting the consumption of local foods could improve the diet quality in African populations.

Teff (*Eragrostis tef* ) is a grain native to Ethiopia and Eritrea (Eastern Africa). Teff is the smallest of all grains and is considered a minor cereal due to its meager use, limited to a few regions of the world (86). Teff is a good source of dietary fiber (8 g/100 g), and it has higher amount of protein (11–13%) as compared to other grains (87). As for micronutrients, teff contains the highest iron and calcium contents (11–33 mg and 100–150 mg, respectively) among all grains (86). Therefore, a cross-sectional study in 592 pregnant women suggested that the lower risk of anemia in Ethiopia might be explained by the daily consumption of this grain (88).

Moringa (*Moringa Adans*) is a plant that can grow in extreme climatic conditions (hot dry and less fertile soils) and is widespread in Ethiopia and Kenya. All botanical parts of Moringa (flowers, pods, leaves, and seeds) have been traditionally used for their potential beneficial effects. Indeed, Moringa is a good source of phytochemicals and micronutrients (89). As for vitamins, Moringa has shown a content of vitamin A and β-carotene (11,300–23,000 IU and 6.6–6.8 mg *per* 100 g), as well as vitamin C (200 mg/100 g), which are even higher than other plant foods (i.e., carrots and pumpkins, and oranges and kiwi, respectively. In addition, the amount of Vitamin E is similar to nuts (9.0 mg/100 g) whereas Moringa contains more polyphenols than fruit and vegetables (88). Pods and seeds contain unsaturated and essential fatty acids, especially oleic acid as well as omega 3, that make Moringa a source of healthy fat (90).

This nutritional profile might contribute to the antioxidant, antimicrobial, and anti-inflammatory activities associated with its consumption. Nevertheless, most of the evidence is provided by *in vitro* experiments and studies in animal models (91). Besides, a recent meta-analysis of 46 studies in animal models suggested a potential role of Moringa-derived products in the reduction of glucose and lipid levels (92).

Native fruit and vegetable, particularly from South Africa, have been reported as relevant contributors to the survival of local communities. It is worth mentioning that almost 119 species of plant-derived products grow in Africa (93). Undoubtedly, fruit and vegetable provide huge amounts of vitamins, minerals, and other phytochemicals with beneficial effects on health.

Recently, Nkosi et al. (94) evaluated the nutritional composition of ten African fruit [*Ficus capensis Thunb* (Cape fig), *Landolphia kirkii* (Sand apricot vine), *Engelerophytum magalismontanum (*Transvaal milkplum*), Parinari curatellifolia* (Mobola plum), *Sclerocarya birrea* (Marula), *Strychnos spinosa* (Green monkey orange), *Strychnos madagascariensis* (Black monkey orange), *Syzgium cordatum* (Water berry), *Ximenia caffra* (Sour plum), and *Vangueria infausta* (Wild medlar)]. They detected different profile of phenolic compounds thus suggesting a potential involvement in different biological activities. Indeed, *in vitro* experiments showed that Mobola plum, Water berry, and Marula presented the strongest antioxidant activity. Conversely, Sour plum and Sand apricot vine) induced the highest inhibition of carbohydrate hydrolysing enzymes, thus suggesting a potential role in carbohydrates digestion.

Unfortunately, scientific evidence in humans or large information on food composition are lacking (93). Nevertheless, the consumption of local fruit and vegetable should be encouraged to improve the diet quality and sustainability of food systems.

In conclusion, to improve diet quality in African populations the proposed changes are:

- favor the consumption of native grains like teff by consuming at least 2 servings/day of teff-derived products
- use Moringa oil as daily fat source (the amount should be according to individual energy needs)
- increase the intake of plant-based foods according to African traditional diet and endorse the use of native fruits (1 or 2 servings/day) and vegetables (at least 2 servings/day).

**Figure 3:**
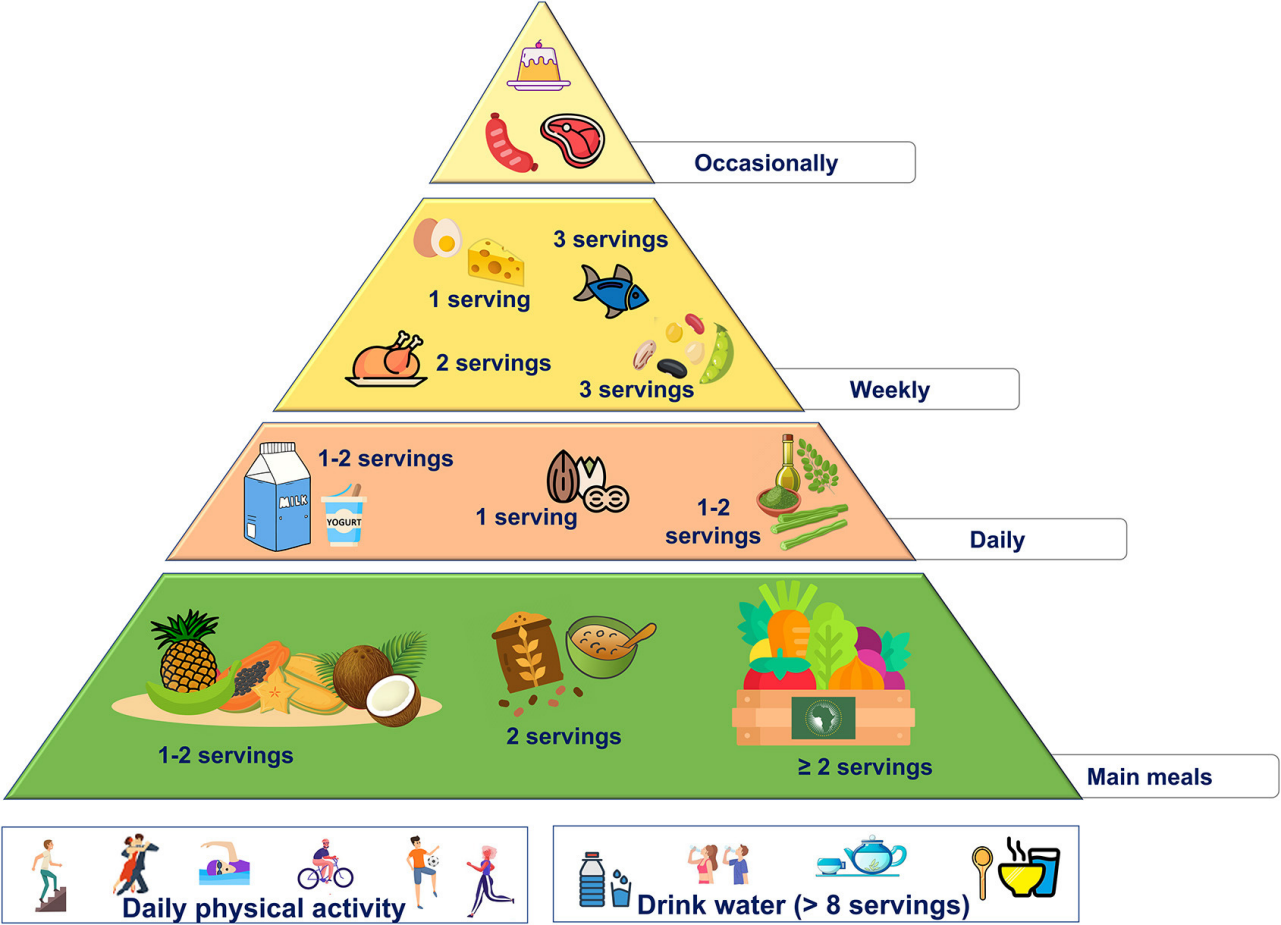
Proposed nutritional pyramid for Africa.

These changes are summarized in the new food pyramid for Africa (Figure 3).

### Asia

The Asian continent is very extended and, consequently, dietary habits might be more heterogeneous across the different countries than in other continents. Although each Asian region has its distinct traditions, there are many unifying characteristics that allows us to perform a critical appraisal of the nutritional profile of population living in Asia.

Grains account for the main part of crops in Asian countries this continent (95–97). Indeed, carbohydrates intake is high (65% to >80% of daily energy intake), with rice and its derivatives being the main dietary sources (98). Although different rice varieties are consumed across Asian countries, white rice is the main contributor to carbohydrate intake. In addition, the higher consumption of rice, the lower intake of fat sources and wholegrain (almost 15 and 5%, respectively) (99). This translates in a high-carbohydrate-low-fat diet characterized by the consumption of food with high glycemic index and glycemic load (99). Several studies have shown that both carbohydrate amount and quality associated with MS and metabolic diseases in Asian countries (100, 101) as well as in other populations (102–104).

Besides, the prevalence of micronutrient deficiencies (mainly vitamin A, vitamin B12, iron, iodine, and folic acid) is widespread in different Asian countries (105).

Therefore, the main dietary changes that should be encouraged are related to the consumption of low-glycemic index starchy foods, adequate carbohydrate intake (<60%), and foodstuffs containing vitamins, minerals, and phytochemicals.

Barley (*Hordeum vulgare L*.) is a major food crop in Middle East (Iran, Iraq, and Syria) and Turkey, but also in Central and South Asia (mainly Kazakhstan, Afghanistan, India, and Pakistan) (46). It is classified as wholegrain, and it received the health claim from its lowering effects on glucose and cholesterol levels by the Food and Drug Administration (FDA) and the European Food Safety Authority (EFSA) (106, 107). These metabolic effects are triggered by β-glucan, a viscous soluble dietary fiber, which can form a viscous gel that can reduces carbohydrate and fat absorption in the gut (108). On the other hand, it is more prone to fermentation by gut microbiota than other type of fibers, thus inducing a greater production of beneficial microbial metabolites (i.e., SCFAs) (109).

Sesame seeds (*Sesamum indicum*) are traditional food in East Asia, but its oil is widely used in the whole continent (110). Sesame is richer in lignans (sesamin, sesamol, sesamolin, episesamin) and γ-tocopherol than other foods (i.e., flaxseeds, nuts, grains, and legumes) (111). In addition, sesame oil contains a beneficial fatty acids profile, consisting of linoleic acid (41%), oleic acid (39%), palmitic acid (8%), stearic acid (5%) (112).

Studies in humans have investigated the possible effects of daily sesame oil consumption on cardiovascular risk factors (113, 114). A meta-analysis of 8 randomized controlled trials (*n* = 843 participants) showed that sesame consumption (35 g of sesame oil) can reduce systolic as well as diastolic blood pressure (−7.83 and −5.83 mmHg, respectively) (113). More recently, Yargholi et al. evaluated the effect of sesame consumption on blood glucose control in a meta-analysis of 8 randomized controlled trials (*n* = 382 participants with T2D). A significant reduction of fasting blood glucose (−28 mg/dl) and HbA1c (−1.00%) levels were observed with the consumption of 30 g of sesame-derived products (114).

Marine macroalgae i.e., seaweeds, have been consumed for centuries in Far East Asia, mainly Japan and Korea (115, 116). They are a great source of complex polysaccharides, minerals, proteins, and vitamins, and well as of several phytochemicals (117, 118). In addition, seaweeds have a significant amount of essential PUFA, namely eicosapentaenoic acid (EPA; 20:5 n-3) and docosahexaenoic acid (DHA; 22:6 n-3), and their precursors α-linolenic acid (ALA; 18:3 n-3) and docosapentaenoic acid (DPA; 22:5 n-3) (119).

The regular consumption of seaweeds (15 g/day) has been associated with a reduced risk of CVD risk (119, 120). This effect is triggered mainly by the lowering-triglyceride effect of DHA and EPA, as stated also by a health claim by EFSA (121).

Nevertheless, some seaweeds have shown to influence other CVD risk factors. Spirulina (*Spirulina platensis*), is a blue-green algae which grows naturally in high salt alkaline water reservoirs in subtropical and tropical areas of Asia. It is a good source of high-quality protein (60–70%/dry weight) and contains an iron with high bioavailability as compared to other foods (i.e., grains) (122). Interestingly, in a metanalyses of randomized controlled trials the sprilunine supplementation decrease total and LDL-cholesterol (−47 and −41 mg/dl, respectively) while increase HDL-cholesterol concentrations (6 mg/dl) (123). Besides, a recent meta-analysis has shown that spiruline consumption (1–8 g) decrease systolic and diastolic blood pressure (−4.59 and −7.02 mmHg, respectively), with greater reduction in patients with hypertension (124).

Wakame (*Undaria pinnatifida*) is one of the most consumed macroalgae worldwide. It contains higher amounts of alginate, a polysaccharide with high viscosity (125). Therefore, its potential role in the modulation of glucose and lipid metabolism has been postulated.

Izaola et al. (126) investigated the effects of a wakame-enriched snack on CVD risk factors in 40 individuals with MS. After 8 weeks, a significant reduction of total and LDL-cholesterol (−10 and −8.9 mg/dl, respectively) observed with no effects on glucose metabolism. More recently, in an acute cross-over trial, wakame intake (5 g) significantly reduced glucose and insulin response to a mixed meal compared to the control (127). However, it is worth mentioning that seaweeds contain iodine their overconsumption may pose a risk of thyroid diseases in susceptible individuals (128). In addition, due to pollution contamination in the aquatic system, seaweeds might contain heavy metals and metalloids (129). Therefore, regular consumption of seaweeds should be monitored due to the potential health risk in the long term.

Soy (*Glycine max*) and its derivatives are largely used in Asian countries. Soy is an East Asian native leguminous plant rich in proteins (364–6%, depending on the variety), lipids (18%), soluble carbohydrates (15%), and fiber (15%). In addition, soy also contains several bioactive compounds such as lecithin (0.5%), sterols (0.3%), isoflavones (0.1%), tocopherols and tocotrienols (0.02%) (130).

Epidemiological studies have shown an inverse association between soy-based foods and the incidence of CVD, T2D, and certain types of cancer (breast and stomach) (131–137). These effects are related to its isoflavone content. Indeed, isoflavones are phytoestrogens which can bind the estrogen receptor thus having an estrogen-like activity (130). Moreover, soy proteins (β-conglycinin and glycinin) and peptides obtained by their intestinal hydrolysis have repeatedly shown a cholesterol-lowering effects by promoting LDL-receptor expression (138). Notably, soy intake (25 g/day) has been granted by FDA for its beneficial effects on cardiovascular health (139).

As reported above, the traditional Asian diet is particularly rich in starchy foods with high glycemic index, and lower amount of other nutrients and possibly micronutrients. Therefore, some traditional foodstuffs should be limited favoring other foodstuff with more beneficial effects on health. Accordingly, the following changes are proposed to improve diet quality:

- 2 servings/day of starchy foods with low glycemic index (barley and wholegrain)
- no more than 2 servings/week of starchy foods with high glycemic index (rice, and noodles)
- use sesame oil as the main daily fat source (the amount should be according to individual energy needs), and use sesame seeds to enrich soups
- increase the intake of vegetables (at least 2 servings/day) and fruits (1–2 servings/day), preferring fruit with high antioxidant activities.
- 2 servings/week of plant protein sources (soy-derived foods)
- 1 serving/day of seaweeds (in particular, spirulina and wakame)

**Figure 4:**
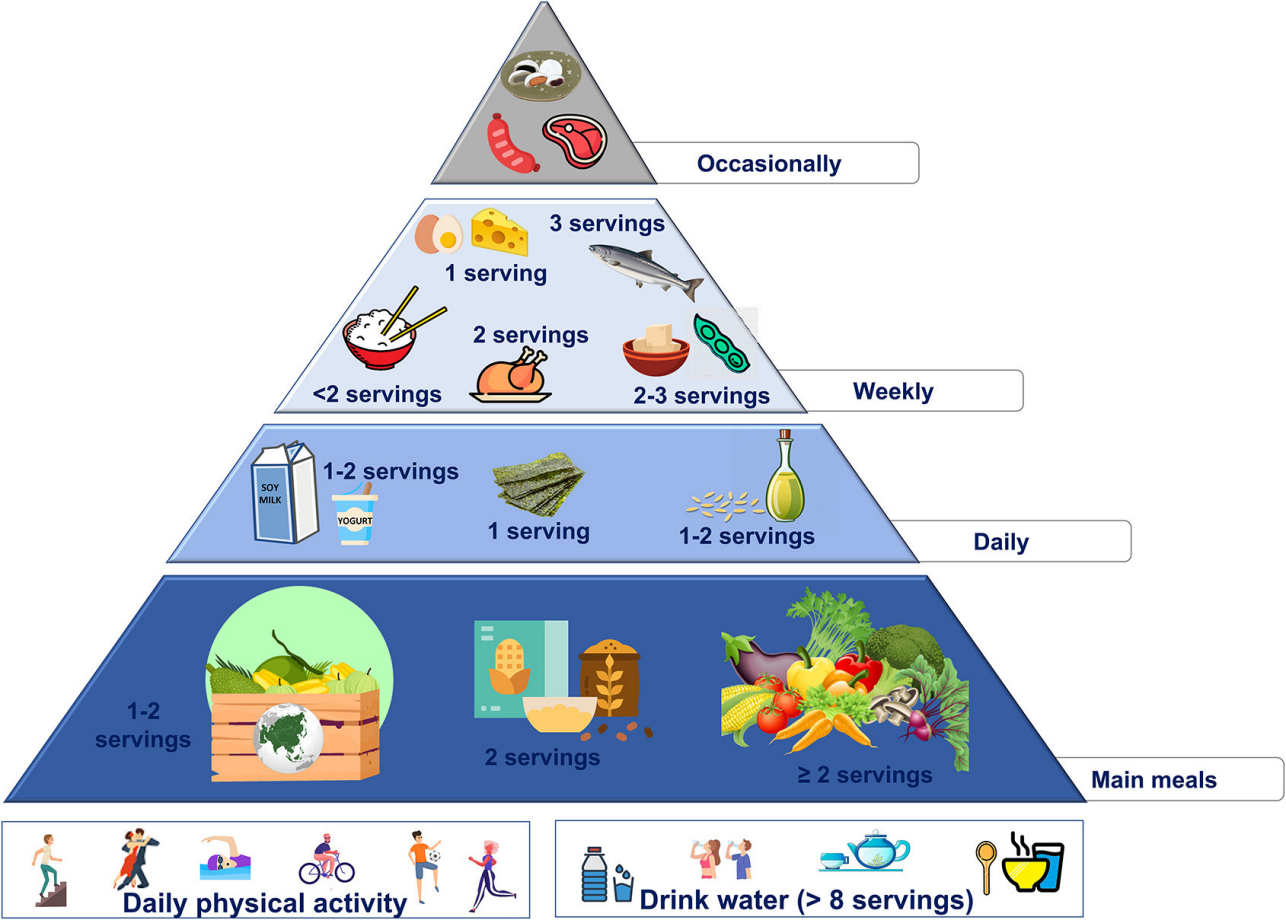
Proposed nutritional pyramid for Asia.

These changes are summarized in the new food pyramid for Asia (Figure 4).

### Australia

The actual Australian diet resembles a Western dietary pattern, with plant-based foods replaced by high-fat, energy-dense, and greater animal-derived foodstuffs (140). Therefore, due to the “nutrition transition,” dietary changes favoring healthier food choices of local products could represent a first step to improve diet quality also in Australia.

Macadamia nut *(Macadamia integrifolia*), a tree nut native to Australia, contains ~75% fat, higher levels of MUFA than any other food sources (more than 60 g/100 g), and phenolic compounds (141).

As for the potential health benefits of macadamia nuts intake, few studies in humans are available. In the study by Garg et al. 17 hypercholesterolemic male were given macadamia nuts (40–90 g/day, accounting for 15% of total energy intake) for 4 weeks. At the end of the intervention, plasma markers of inflammation (leukotriene) and oxidative stress (8-isoprostane) were significantly lower in the group consuming macadamia nuts than control (leukotriene: −323 ± 96 pg/ml and 8-isoprostane: 197 ± 19 pg/ml) (142). In addition, in a 5-week clinical trial with macadamia nut supplementation (40–90 g/day, accounting for 15% of total energy intake) significantly reduced total and LDL-cholesterol as compared to control (−20 ± 7 and −12 ± 5 mg/dl, respectively) in a group of 25 hypercholesterolemic men and women (143).

Atlantic salmon (*Salmo salar*) and Barramundi (*Lates calcarifer*) are two major Australian farmed fish species. They are rich in omega-3 PUFA with 980 and 790 mg/100 g, respectively (144). As reported above, omega 3 fatty acids (namely DHA and EPA) bear a health claim for their triglyceride-lowering effects (121), possibly contributing to the reduction of CVD risk.

Native fruits, i.e., Davidson's plum (*Davidsonia spp*.), pepper berry (*Tasmannia lanceolata*), finger lime (*Citrus australasica var. sanguinea)*, are great contributors for dietary intake of vitamins, minerals, and other phytochemicals with beneficial effects on health (145).

Preliminary studies suggested that the extracts of these fruits might inhibit cancer cells growth in several *in vitro* models (pancreas, breast, lung, brain, skin, colon, and ovary cancers) (146, 147). Unfortunately, to the best of our knowledge, no clinical studies in humans are available so far.

As reported above, important changes are required to improve the overall quality of the actual Australian diet. However, to increase the feasibility of these dietary changes, the incorporation of local foods rich in fiber, phytochemical and other bioactive compounds may represent a complementary strategy. Therefore, over the main principles of the MD for animal protein-sources and sweets, the following changes are proposed:

- use macadamia oil as the main daily fat source (the amount should be according to individual energy needs)
- increase the intake of vegetables (at least 2 servings/day), preferring local varieties
- increase the intake of fruits (>2 servings/day), preferring local varieties (i.e., Davidson's plum, native pepper berry, and finger lime)
- increase the intake of fish rich in omega-3 PUFA (2–3 servings/week), preferring local varieties (i.e., Atlantic salmon, barramundi)
- use macadamia nuts for snacking, in particular (40–90 g/day, accounting for 15% of total energy intake).

**Figure 5:**
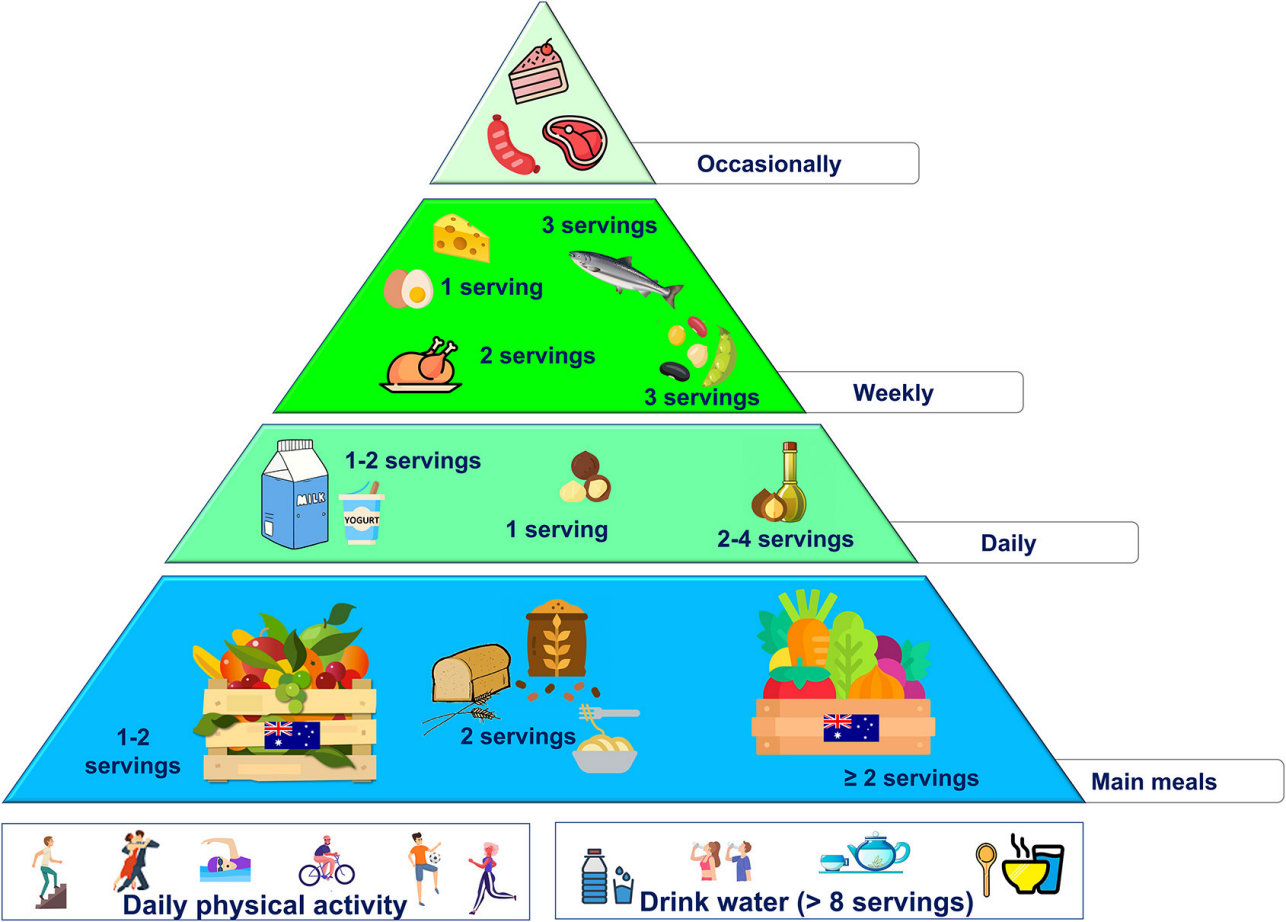
Proposed nutritional pyramid for Australia.

These changes are summarized in the new food pyramid for Australia (Figure 5).

## Conclusion and future perspectives

Although the pivotal role of the MD in the prevention and management of NCDs, it is not easy to transfer this dietary pattern to other populations. Indeed, the adoption of the real MD implies to shift from local traditions that have been in those territories for centuries to new and unknown habits. Therefore, it seems more reliable—and also desirable- that each country rediscovers its own heritage to develop a healthier nutrition pattern based on traditional and local foods. This would be aligned with UNESCO advocacy to preserve cultural identity, continuity of communities, and environment.

Some non-Mediterranean dietary patterns, as Nordic (148) and Okinawan (149) diets, have been shown to reduce disease risk and mortality. These beneficial effects are linked to a nutritional profile that is superimposable to MD (150, 151). Therefore, it would be possible to obtain healthful effects by combining different foodstuff belonging to the own country with significant advantages not only for people but also for the environment.

The present review is an attempt to promote a healthy and sustainable dietary model based on the nutritional properties of MD but implemented at the local level by using the food products available in different areas of the world. Nevertheless, as the first release of the research project “Planeterranea,” this manuscript provides an overview of the rationale, aims, and main results, and the limitations will be addressed in future publications. Indeed, some of the analyzed macro-areas (Asia, Africa) include many heterogenous countries with specific eating habits. Therefore, it is necessary to evaluate whether a macro-area might require multiple nutritional pyramids to endorse the compliance of the different populations.

On the other hand, the sustainability of the proposed pyramids should be evaluated in *ad hoc* investigations that should consider (a) affordability in terms of commercialization, costs, farming, etc.; (b) impact on local economies that should meet the needs of growing populations; (c) greenhouse gas emissions of local crops as compared to long-traveling food products.

Finally, more studies are needed to evaluate the bioavailability of micronutrients and bioactive compounds contained in the identified foods. In addition, the safety of local foods with potential health benefits might be investigated to assess whether they might have detrimental side effects in the long term.

In conclusion, it is important to increase global knowledge about healthy and sustainable dietary patterns rather than force people to a useless change. Therefore, nutritional research should focus not only on the amount and frequency of food consumed but also on cultural behaviors, socio-economic conditions, food quality and processing. This would lead to a sharply increased adherence to nutritional recommendations with a relevant impact on public health and clinical practice.

## Author contributions

CV, PP, and AC: conceptualization and validation. CV and PP: writing—original draft preparation. GM, LB, DL, CG, and FM: review and editing. AC: supervision. All authors have read and agreed to the published version of the manuscript.

## Funding

This research was funded by PRIN from Ministry of University and Research, Grant PRIN3292020NCKXBR (to AC), entitled “Suscettibilità alle malattie infettive nell’obesità: una valutazione endocrina, traslazionale e sociologica (SIDERALE)”.

## Conflict of interest

The authors declare that the research was conducted in the absence of any commercial or financial relationships that could be construed as a potential conflict of interest.

## Publisher's note

All claims expressed in this article are solely those of the authors and do not necessarily represent those of their affiliated organizations, or those of the publisher, the editors and the reviewers. Any product that may be evaluated in this article, or claim that may be made by its manufacturer, is not guaranteed or endorsed by the publisher.

## References

[1] GBD 2019 Diseases and Injuries Collaborators Global burden of 369 diseases and injuries in 204 countries and territories 1990–2019: 1990–2019: a systematic analysis for the Global Burden of Disease Study 2019. Lancet (London, England). (2020) 396(10258):1204–22. 10.1016/S0140-6736(20)30925-9 33069326 PMC7567026

[2] Lin X Xu Y Pan X Xu J Ding Y Sun X. Global, regional, and national burden and trend of diabetes in 195 countries and territories: an analysis from 1990 to 2025. Sci Rep. (2020) 10:14790. 10.1038/s41598-020-71908-9 32901098 PMC7478957

[3] Chen H Chen G Zheng X Guo Y. Contribution of specific diseases and injuries to changes in health adjusted life expectancy in 187 countries from 1990 to 2013: retrospective observational study. BMJ (Clinical research ed.). (2019) 364:l969. 10.1136/bmj.l969 30917970 PMC6435998

[4] Safiri S Karamzad N Singh K Carson-Chahhoud K Adams C Nejadghaderi SA. Burden of ischemic heart disease and its attributable risk factors in 204 countries and territories, 1990-2019. Eur J Prev Cardiol. (2022) 29:420–431. 10.1093/eurjpc/zwab213 34922374

[5] Global Burden of Disease 2019 Cancer Collaboration Kocarnik JM Compton K Dean FE Fu W Gaw BL. Cancer incidence, mortality, years of life lost, years lived with disability, and disability-adjusted life years for 29 cancer groups from 2010 to 2019: a systematic analysis for the global burden of disease study 2019. JAMA Oncol. (2022) 8:420–44. 10.1001/jamaoncol.2021.6987 34967848 PMC8719276

[6] Armocida B Monasta L Sawyer S Bustreo F Segafredo G Castelpietra G. Burden of non-communicable diseases among adolescents aged 10-24 years in the EU, 1990-2019: a systematic analysis of the Global Burden of Diseases Study 2019. Lancet Child Adolesc Health. (2022) 6:367–83. 10.1016/S2352-4642(22)00073-6 35339209 PMC9090900

[7] Liu L Villavicencio F Yeung D Perin J Lopez G Strong KL. National, regional, and global causes of mortality in 5–19-year-olds from 2000 to 2019: a systematic analysis. Lancet Glob Health. (2022) 10:e337–47. 10.1016/S2214-109X(21)00566-0 35180417 PMC8864304

[8] GBD 2017 Diet Collaborators. Health effects of dietary risks in 195 countries, 1990-2017: a systematic analysis for the Global Burden of Disease Study 2017. Lancet (London, England). (2019) 393:1958–72. 10.1016/S0140-6736(19)30041-8 30954305 PMC6899507

[9] Lozano R Naghavi M Foreman K Lim S Shibuya K Aboyans V. Global and regional mortality from 235 causes of death for 20 age groups in 1990 and 2010: a systematic analysis for the Global Burden of Disease Study 2010. Lancet (London, England). (2012) 380:2095–128. 10.1016/S0140-6736(12)61728-0 23245604 PMC10790329

[10] Safiri S Karamzad N Kaufman JS Nejadghaderi SA Bragazzi NL Sullman MJM. Global, regional, and national burden of cancers attributable to excess body weight in 204 countries and territories, 1990 to 2019. Obesity (Silver Spring, Md.). (2022) 30:535–45. 10.1002/oby.23355 35041300

[11] Shai I Schwarzfuchs D Henkin Y Shahar DR Witkow S Greenberg I. Weight loss with a low-carbohydrate, Mediterranean, or low-fat diet. N Engl J Med. (2008) 359:229–41. 10.1056/NEJMoa0708681 18635428

[12] Mancini JG Filion KB Atallah R Eisenberg MJ. Systematic review of the mediterranean diet for long-term weight loss. Am J Med. (2016) 129:407–15. 10.1016/j.amjmed.2015.11.028 26721635

[13] Grosso G Marventano S Yang J Micek A Pajak A Scalfi L. A comprehensive meta-analysis on evidence of Mediterranean diet and cardiovascular disease: Are individual components equal? Crit Rev Food Sci Nutr. (2017) 57:3218–32. 10.1080/10408398.2015.1107021 26528631

[14] Sotos-Prieto M Bhupathiraju SN Mattei J Fung TT Li Y Pan A. Changes in diet quality scores and risk of cardiovascular disease among US men and women. Circulation. (2015) 132:2212–9. 10.1161/CIRCULATIONAHA.115.017158 26644246 PMC4673892

[15] Schwingshackl L Missbach B König J Hoffmann G. Adherence to a Mediterranean diet and risk of diabetes: a systematic review and meta-analysis. Public Health Nutr. (2015) 18:1292–9. 10.1017/S1368980014001542 25145972 PMC10273006

[16] Altieri B Barrea L Modica R Muscogiuri G Savastano S Colao A. Nutrition and neuroendocrine tumors: an update of the literature. Rev Endocr Metab Disord. (2018) 19:159–67. 10.1007/s11154-018-9466-z 30267297

[17] Barrea L Altieri B Muscogiuri G Laudisio D Annunziata G Colao A. Impact of nutritional status on gastroenteropancreatic neuroendocrine tumors (GEP-NET) aggressiveness. Nutrients. (2018) 10:1854. 10.3390/nu10121854 30513732 PMC6316835

[18] Laudisio D Barrea L Muscogiuri G Annunziata G Colao A Savastano S. Breast cancer prevention in premenopausal women: role of the Mediterranean diet and its components. Nutr Res Rev. (2020) 33:19–32. 10.1017/S0954422419000167 31571551

[19] Barrea L Pugliese G Frias-Toral E Laudisio D Rodriguez D Vitale G. Diet as a possible influencing factor in thyroid cancer incidence: the point of view of the nutritionist. Panminerva Med. (2021) 63:349–60. 10.23736/S0031-0808.21.04213-0 33878846

[20] Laudisio D Castellucci B Barrea L Pugliese G Savastano S Colao A et al. Mediterranean diet and breast cancer risk: a narrative review. Minerva Endocrinol (Torino). (2021) 46:441–52. 10.23736/S2724-6507.20.03266-6 32969630

[21] Mazzocchi A Leone L Agostoni C Pali-Schöll I. The secrets of the mediterranean diet: Does [Only] Olive Oil Matter? Nutrients. (2019) 11:2941. 10.3390/nu11122941 31817038 PMC6949890

[22] Vassilopoulou E Guibas GV Papadopoulos NG. Mediterranean-type diets as a protective factor for asthma and atopy. Nutrients. (2022) 14:1825. 10.3390/nu14091825 35565792 PMC9105881

[23] Bach-Faig A Berry EM Lairon D Reguant J Trichopoulou A Dernini S. Mediterranean diet pyramid today. science and cultural updates. Public Health Nutr. (2011) 14:2274–84. 10.1017/S1368980011002515 22166184

[24] Barrea L Pugliese G Laudisio D Colao A Savastano S Muscogiuri G. Mediterranean diet as medical prescription in menopausal women with obesity: a practical guide for nutritionists. Crit Rev Food Sci Nutr. (2021) 61:1201–11. 10.1080/10408398.2020.1755220 32329636

[25] Barrea L Muscogiuri G Frias-Toral E Laudisio D Pugliese G Castellucci B. Nutrition and immune system: from the Mediterranean diet to dietary supplementary through the microbiota. Crit Rev Food Sci Nutr. (2021) 61:3066–90. 10.1080/10408398.2020.1792826 32691606

[26] Scannell N Villani A Mantzioris E Swanepoel L. Understanding the self-perceived barriers and enablers toward adopting a mediterranean diet in australia: an application of the theory of planned behavior framework. Int J Environ Res Public Health. (2020) 17:9321. 10.3390/ijerph17249321 33322111 PMC7764290

[27] Moore SE McEvoy CT Prior L Lawton J Patterson CC Kee F. Barriers to adopting a Mediterranean diet in Northern European adults at high risk of developing cardiovascular disease. J Hum Nutr Diet. (2018) 31:451–462. 10.1111/jhn.12523 29159932

[28] HLPE. Food security and nutrition: building a global narrative toward 2030. A report by the High Level Panel of Experts on Food Security and Nutrition of the Committee on World Food Security, Rome (2020).

[29] Willett W Rockström J Loken B. The EAT-Lancet commission: a flawed approach?—Authors' reply. Lancet. (2019) 394:1141–2. 10.1016/S0140-6736(19)31910-5 31571599

[30] UNESCO, Chair “Federico II,.” https://www.unescochairnapoli.it/?lang=en (accessed 30/12/2021).

[31] Shan Z Rehm CD Rogers G Ruan M Wang DD Hu FB. Trends in dietary carbohydrate, protein, and fat intake and diet quality among US Adults, 1999-2016. JAMA. (2019) 322:1178–87. 10.1001/jama.2019.13771 31550032 PMC6763999

[32] Martínez-González MÁ Hershey MS Zazpe I Trichopoulou A. Transferability of the mediterranean diet to non-mediterranean countries. what is and what is not the mediterranean diet. Nutrients. (2017) 9:1226. 10.3390/nu9111226 29117146 PMC5707698

[33] Iggman D Gustafsson IB Berglund L Vessby B Marckmann P Risérus U. Replacing dairy fat with rapeseed oil causes rapid improvement of hyperlipidaemia: a randomized controlled study. J Intern Med. (2011) 270:356–64. 10.1111/j.1365-2796.2011.02383.x 21466598

[34] Kanikowska D Kanikowska A Rutkowski R Włochal M Orzechowska Z Juchacz A. Amaranth (Amaranthus cruentus L.) and canola (Brassica napus L.) oil impact on the oxidative metabolism of neutrophils in the obese patients. Pharm Biol. (2019) 57:140–4. 10.1080/13880209.2019.1569696 30905230 PMC6442228

[35] Ghobadi S Hassanzadeh-Rostami Z Mohammadian F Zare M Faghih S. Effects of canola oil consumption on lipid profile: a systematic review and meta-analysis of randomized controlled clinical trials. J Am Coll Nutr. (2019) 38:185–96. 10.1080/07315724.2018.1475270 30381009

[36] Amiri M Raeisi-Dehkordi H Sarrafzadegan N Forbes SC Salehi-Abargouei A. The effects of Canola oil on cardiovascular risk factors: a systematic review and meta-analysis with dose-response analysis of controlled clinical trials. Nutr Metab Cardiovasc Dis. (2020) 30:2133–45. 10.1016/j.numecd.2020.06.007 33127255

[37] Raeisi-Dehkordi H Amiri M Humphries KH Salehi-Abargouei A. The effect of canola oil on body weight and composition: a systematic review and meta-analysis of randomized controlled clinical trials. Adv Nutr. (2019) 10:419–32. 10.1093/advances/nmy108 30809634 PMC6520036

[38] Morgan WA Clayshulte BJ. Pecans lower low-density lipoprotein cholesterol in people with normal lipid levels. J Am Diet Assoc. (2000) 100:312–8. 10.1016/S0002-8223(00)00097-3 10719404

[39] Bolling BW Chen CY McKay DL Blumberg JB. Tree nut phytochemicals: composition, antioxidant capacity, bioactivity, impact factors: a systematic review of almonds, brazils, cashews, hazelnuts, macadamias, pecans, pine nuts, pistachios and walnuts. Nutr Res Rev. (2011) 24:244–75. 10.1017/S095442241100014X 22153059

[40] Rajaram S Burke K Connell B Myint T Sabaté J. A monounsaturated fatty acid-rich pecan-enriched diet favorably alters the serum lipid profile of healthy men and women. J Nutr. (2001) 131:2275–9. 10.1093/jn/131.9.2275 11533266

[41] Campos VP Portal VL Markoski MM Quadros AS Bersch-Ferreira ÂC Garavaglia J. Effects of a healthy diet enriched or not with pecan nuts or extra-virgin olive oil on the lipid profile of patients with stable coronary artery disease: a randomized clinical trial. J Hum Nutr Diet. (2020) 33:439–50. 10.1111/jhn.12727 31856379

[42] McKay DL Eliasziw M Chen CYO Blumberg JB. A pecan-rich diet improves cardiometabolic risk factors in overweight and obese adults: a randomized controlled trial. Nutrients. (2018) 10:339. 10.3390/nu10030339 29534487 PMC5872757

[43] Romdhane MH Chahdoura H Barros L Dias MI Carvalho Gomes Corrêa R Morales P. Chemical composition, nutritional value, and biological evaluation of tunisian okra pods (Abelmoschus esculentus L. Moench). Molecules. (2020) 25:4739. 10.3390/molecules25204739 33076530 PMC7587556

[44] Durazzo A Lucarini M Novellino E Souto EB Daliu P Santini A. Abelmoschus esculentus (L.): bioactive components' beneficial properties-focused on antidiabetic role-for sustainable health applications. Molecules. (2018) 24:38. 10.3390/molecules24010038 30583476 PMC6337517

[45] Elkhalifa AEO Alshammari E Adnan M Alcantara JC Awadelkareem AM Eltoum NE. Okra (*Abelmoschus Esculentus*) as a potential dietary medicine with nutraceutical importance for sustainable health applications. Molecules. (2021) 26:696. 10.3390/molecules26030696 33525745 PMC7865958

[46] USDA. Available online at: https://ipad.fas.usda.gov/ogamaps/cropproductionmaps.aspx, (accessed 30/12/2021).

[47] Mullins AP Arjmandi BH. Health benefits of plant-based nutrition: focus on beans in cardiometabolic diseases. Nutrients. (2021) 13:519. 10.3390/nu13020519 33562498 PMC7915747

[48] Winham DM Hutchins AM Johnston CS. Pinto bean consumption reduces biomarkers for heart disease risk. J Am Coll Nutr. (2007) 26:243–9. 10.1080/07315724.2007.10719607 17634169

[49] NCEP. Expert Panel on Detection, Evaluation, and Treatment of High Blood Cholesterol in Adults (Adult Treatment Panel III). Third Report of the National Cholesterol Education Program (NCEP) Expert Panel on Detection, Evaluation, and Treatment of High Blood Cholesterol in Adults (Adult Treatment Panel III) final report. Circulation. (2002) 106:3143–21. 10.1161/circ.106.25.3143 12485966

[50] Finley JW Burrell JB Reeves PG. Pinto bean consumption changes SCFA profiles in fecal fermentations, bacterial populations of the lower bowel, and lipid profiles in blood of humans. J Nutr. (2007) 137:2391–8. 10.1093/jn/137.11.2391 17951475

[51] Kovalskys I Fisberg M Gómez G Pareja RG Yépez García MC Cortés Sanabria LY. Energy intake and food sources of eight Latin American countries: results from the Latin American Study of Nutrition and Health (ELANS). Public Health Nutr. (2018) 21:2535–47. 10.1017/S1368980018001222 29848396 PMC10260935

[52] Gómez G Fisberg RM Nogueira Previdelli Á Hermes Sales C Kovalskys I Fisberg M. Diet quality and diet diversity in eight Latin American countries: results from the latin American study of nutrition and health (ELANS). Nutrients. (2019) 11:1605. 10.3390/nu11071605 31311159 PMC6682987

[53] Jaacks LM Vandevijvere S Pan A McGowan CJ Wallace C Imamura F. The obesity transition: stages of the global epidemic. Lancet Diabetes Endocrinol. (2019) 7:231–240. 10.1016/S2213-8587(19)30026-9 30704950 PMC7360432

[54] Angeli V Miguel Silva P Crispim Massuela D Khan MW Hamar A Khajehei F. Quinoa (Chenopodium quinoa Willd.): an overview of the potentials of the “golden grain” and socio-economic and environmental aspects of its cultivation and marketization. Foods. (2020) 9:216. 10.3390/foods9020216 32092899 PMC7074363

[55] Gordillo-Bastidas E Díaz-Rizzolo DA Roura E Massanés T Gomis R. Quinoa (Chenopodium quinoa Willd), from nutritional value to potential health benefits: an integrative review. J Nutr Food Sci. (2016) 6:3

[56] Vega-Gálvez A Miranda M Vergara J Uribe E Puente L Martínez EA. Nutrition facts and functional potential of quinoa (Chenopodium quinoa willd.), an ancient Andean grain: a review. J Sci Food Agric. (2010) 90:2541–547. 10.1002/jsfa.4158 20814881

[57] Martínez-Villaluenga C Peñas E Hernández-Ledesma B. Pseudocereal grains: nutritional value, health benefits and current applications for the development of gluten-free foods. Food Chem Toxicol. (2020) 137:111178. 10.1016/j.fct.2020.111178 32035214

[58] Filho AM Pirozi MR Borges JT Pinheiro Sant'Ana HM Chaves JB Coimbra JS. Quinoa: nutritional, functional, and antinutritional aspects. Crit Rev Food Sci Nutr. (2017) 57:1618–30. 10.1080/10408398.2014.1001811 26114306

[59] Graf BL Rojas-Silva P Rojo LE Delatorre-Herrera J Baldeón ME Raskin I. Innovations in health value and functional food development of Quinoa (Chenopodium quinoa Willd). Compr Rev Food Sci Food Saf. (2015) 14:431–45. 10.1111/1541-4337.12135 27453695 PMC4957693

[60] Karimian J Abedi S Shirinbakhshmasoleh M Moodi F Moodi V Ghavami A. The effects of quinoa seed supplementation on cardiovascular risk factors: a systematic review and meta-analysis of controlled clinical trials. Phytother Res. (2021) 35:1688–96. 10.1002/ptr.6901 33037704

[61] Li L Lietz G Bal W Watson A Morfey B Seal C. Effects of Quinoa (Chenopodium quinoa willd.) consumption on markers of CVD Risk. Nutrients. (2018) 10:777. 10.3390/nu10060777 29914146 PMC6024323

[62] López-Cervantes J Sánchez-Machado D de la Mora-López DS Sanches-Silva A. Quinoa (Chenopodium quinoa Willd.): exploring a superfood from Andean indigenous cultures with potential to reduce cardiovascular disease (CVD) risk markers. Curr Mol Pharmacol. (2021) 14:925–34. 10.2174/1874467214999210111222233 33430757

[63] Alexander C Swanson KS Fahey GC Garleb KA. Perspective: physiologic importance of short-chain fatty acids from non-digestible carbohydrate fermentation. Adv Nutr. (2019) 10:576–89. 10.1093/advances/nmz004 31305907 PMC6628845

[64] Falcomer AL Riquette RFR de Lima BR Ginani VC Zandonadi RP. Health benefits of green banana consumption: a systematic review. Nutrients. (2019) 11:1222. 10.3390/nu11061222 31146437 PMC6627159

[65] Wang Y Chen J Song YH Zhao R Xia L Chen Y. Effects of the resistant starch on glucose, insulin, insulin resistance, and lipid parameters in overweight or obese adults: a systematic review and meta-analysis. Nutr Diabetes. (2019) 9:19. 10.1038/s41387-019-0086-9 31168050 PMC6551340

[66] Gao C Rao M Huang W Wan Q Yan P Long Y. Resistant starch ameliorated insulin resistant in patients of type 2 diabetes with obesity: a systematic review and meta-analysis. Lipids Health Dis. (2019) 18:205. 10.1186/s12944-019-1127-z 31760943 PMC6875042

[67] Deehan EC Yang C Perez-Muñoz ME Nguyen NK Cheng CC Triador L. Precision microbiome modulation with discrete dietary fiber structures directs short-chain fatty acid production. Cell Host Microbe. (2020) 27:389–404. 10.1016/j.chom.2020.01.006 32004499

[68] Hughes RL Horn WH Finnegan P Newman JW Marco ML Keim NL. Resistant starch type 2 from wheat reduces postprandial glycemic response with concurrent alterations in gut microbiota composition. Nutrients. (2021) 13:645. 10.3390/nu13020645 33671147 PMC7922998

[69] Tavares da Silva S Araújo Dos Santos C Marvila Girondoli Y Mello de Azeredo L Fernando de Sousa Moraes L Keila Viana Gomes Schitini J. Women with metabolic syndrome improve antrophometric and biochemical parameters with green banana flour consumption. Nutr Hosp. (2014) 29:1070–80. 10.3305/nh.2014.29.5.7331 24951987

[70] Ble-Castillo JL Aparicio-Trápala MA Francisco-Luria MU Córdova-Uscanga R Rodríguez-Hernández A Méndez JD. Effects of native banana starch supplementation on body weight and insulin sensitivity in obese type 2 diabetics. Int J Environ Res Public Health. (2010) 7:1953–62. 10.3390/ijerph7051953 20623003 PMC2898027

[71] Costa ES França CN Fonseca FAH Kato JT Bianco HT Freitas TT. Beneficial effects of green banana biomass consumption in patients with pre-diabetes and type 2 diabetes: a randomized controlled trial. Br J Nutr. (2019) 121:1365–75. 10.1017/S0007114519000576 30887937

[72] Dreher ML Davenport AJ. Hass avocado composition and potential health effects. Crit Rev Food Sci Nutr. (2013) 53:738–50. 10.1080/10408398.2011.556759 23638933 PMC3664913

[73] USDA (U.S. Department of Agriculture). Department of *Avocado, Almond, Pistachio Walnut Composition. Nutrient Data Laboratory*. USDA National Nutrient Database for Standard Reference, Release 24. U.S. Agriculture. Washington, DC. (2011).

[74] Peou S Milliard-Hasting B Shah SA. Impact of avocado-enriched diets on plasma lipoproteins: a meta-analysis. J Clin Lipidol. (2016) 10:161–71. 10.1016/j.jacl.2015.10.011 26892133

[75] Mahmassani HA Avendano EE Raman G Johnson EJ. Avocado consumption and risk factors for heart disease: a systematic review and meta-analysis. Am J Clin Nutr. (2018) 107:523–36. 10.1093/ajcn/nqx078 29635493

[76] Schoeneck M Iggman D. The effects of foods on LDL cholesterol levels: a systematic review of the accumulated evidence from systematic reviews and meta-analyses of randomized controlled trials. Nutr Metab Cardiovasc Dis. (2021) 31:1325–38. 10.1016/j.numecd.2020.12.032 33762150

[77] Wang L Tao L Hao L Stanley TH Huang KH Lambert JD. A moderate-fat diet with one avocado per day increases plasma antioxidants and decreases the oxidation of small, dense ldl in adults with overweight and obesity: a randomized controlled trial. J Nutr. (2020) 150:276–84. 10.1093/jn/nxz231 31616932 PMC7373821

[78] Edwards CG Walk AM Thompson SV Reeser GE Erdman JW Jr Burd NA. Effects of 12-week avocado consumption on cognitive function among adults with overweight and obesity. Int J Psychophysiol. (2020) 148:13–24. 10.1016/j.ijpsycho.2019.12.006 31846631

[79] Neri-Numa IA Soriano Sancho RA Pereira APA Pastore GM. Small Brazilian wild fruits: Nutrients, bioactive compounds, health-promotion properties and commercial interest. Food Res Int. (2018) 103:345–60. 10.1016/j.foodres.2017.10.053 29389624

[80] Udani JK Singh BB Singh VJ Barrett ML. Effects of Açai (Euterpe oleracea Mart.) berry preparation on metabolic parameters in a healthy overweight population: a pilot study. Nutr J. (2011) 10:45. 10.1186/1475-2891-10-45 21569436 PMC3118329

[81] Barbosa PO Pala D Silva CT de Souza MO do Amaral JF Vieira RA. Açai (Euterpe oleracea Mart.) pulp dietary intake improves cellular antioxidant enzymes and biomarkers of serum in healthy women. Nutrition. (2016) 32:674–80. 10.1016/j.nut.2015.12.030 26883870

[82] Aranha LN Silva MG Uehara SK Luiz RR Nogueira Neto JF Rosa G. Effects of a hypoenergetic diet associated with açaí (Euterpe oleracea Mart.) pulp consumption on antioxidant status, oxidative stress and inflammatory biomarkers in overweight, dyslipidemic individuals. Clin Nutr. (2020) 39:1464–69. 10.1016/j.clnu.2019.06.008 31307842

[83] Laar AK Addo P Aryeetey R Agyemang C Zotor F Asiki G. Perspective: food environment research priorities for africa-lessons from the africa food environment research network. Adv Nutr. (2022) 13:739–47. 10.1093/advances/nmac019 35254411 PMC9156374

[84] Garcia-Closas R Berenguer A González CA. Changes in food supply in Mediterranean countries from 1961 to 2001. Public Health Nutr. (2006) 9:53–60. 10.1079/PHN2005757 16480534

[85] Vila-Real C Pimenta-Martins A Gomes AM Pinto E Maina NH. How dietary intake has been assessed in African countries? A systematic review. Crit Rev Food Sci Nutr. (2018) 58:1002–22. 10.1080/10408398.2016.1236778 27996293

[86] Saturni L Ferretti G Bacchetti T. The gluten-free diet: safety and nutritional quality. Nutrients. (2010) 2:16–34. 10.3390/nu2010016 22253989 PMC3257612

[87] Zhu F. Chemical composition and food uses of teff (Eragrostis tef). Food Chem. (2018) 239:402–15. 10.1016/j.foodchem.2017.06.101 28873585

[88] Mohammed SH Taye H Sissay TA Larijani B Esmaillzadeh A. Teff consumption and anemia in pregnant Ethiopian women: a case-control study. Eur J Nutr. (2019) 58:2011–8. 10.1007/s00394-018-1759-1 29936535

[89] Leone A Spada A Battezzati A Schiraldi A Aristil J Bertoli S. Cultivation, genetic, ethnopharmacology, phytochemistry and pharmacology of moringa oleifera leaves: an overview. Int J Mol Sci. (2015) 16:12791–835. 10.3390/ijms160612791 26057747 PMC4490473

[90] Trigo C Castelló ML Ortolá MD García-Mares FJ Desamparados Soriano M. Moringa oleifera: an unknown crop in developed countries with great potential for industry and adapted to climate change. Foods. (2020) 10:31. 10.3390/foods10010031 33374455 PMC7824577

[91] Kou X Li B Olayanju JB Drake JM Chen N. Nutraceutical or pharmacological potential of moringa oleifera lam. Nutrients. (2018) 10:343. 10.3390/nu10030343 29534518 PMC5872761

[92] Watanabe S Okoshi H Yamabe S Shimada M. Moringa oleifera Lam. in diabetes mellitus: a systematic review and meta-analysis. Molecules. (2021) 26:3513. 10.3390/molecules26123513 34207664 PMC8229498

[93] Van Wyk B.E. The potential of South African plants in the development of new food and beverage products. South African J Bot. (2011) 77:857–868. 10.1016/j.sajb.2011.08.00329427636

[94] Nkosi NJ Shoko T Manhivi VE Slabbert RM Sultanbawa Y Sivakumar D. Metabolomic and chemometric profiles of ten southern African indigenous fruits. Food Chem. (2022) 381:132244. 10.1016/j.foodchem.2022.132244 35184010

[95] Shimazu T Kuriyama S Hozawa A Ohmori K Sato Y Nakaya N. Dietary patterns and cardiovascular disease mortality in Japan: a prospective cohort study. Int J Epidemiol. (2007) 36:600–9. 10.1093/ije/dym005 17317693

[96] Wei X Yu D Ju L Cheng X Zhao L. Analysis of the correlation between eating away from home and bmi in adults 18 years and older in China: data from the CNNHS 2015. Nutrients. (2021) 14:146. 10.3390/nu14010146 35011020 PMC8747186

[97] Wang Y Dai Y Tian T Zhang J Xie W Pan D. The effects of dietary pattern on metabolic syndrome in jiangsu province of China: based on a nutrition and diet investigation project in Jiangsu Province. Nutrients. (2021) 13:4451. 10.3390/nu13124451 34960003 PMC8708757

[98] Tan D Olden AN Orengo A Francey C Campos VC Fayet-Moore F. An assessment of three carbohydrate metrics of nutritional quality for packaged foods and beverages in Australia and Southeast Asia. Nutrients. (2020) 12:2771. 10.3390/nu12092771 32932799 PMC7551443

[99] Lee YJ Song S Song Y. High-carbohydrate diets and food patterns and their associations with metabolic disease in the Korean population. Yonsei Med J. (2018) 59:834–842. 10.3349/ymj.2018.59.7.834 30091316 PMC6082982

[100] Feng R Du S Chen Y Zheng S Zhang W Na G. High carbohydrate intake from starchy foods is positively associated with metabolic disorders: a Cohort Study from a Chinese population. Sci Rep. (2015) 5:16919. 10.1038/srep16919 26581652 PMC4652281

[101] Ha K Kim K Chun OK Joung H Song Y. Differential association of dietary carbohydrate intake with metabolic syndrome in the US and Korean adults: data from the 2007-2012 NHANES and KNHANES. Eur J Clin Nutr. (2018) 72:848–860. 10.1038/s41430-017-0031-8 29339830

[102] Dehghan M Mente A Zhang X Swaminathan S Li W. Prospective Urban Rural Epidemiology (PURE) study investigators. Associations of fats and carbohydrate intake with cardiovascular disease and mortality in 18 countries from five continents (PURE): a prospective cohort study. Lancet. (2017) 390:2050–62. 10.1016/S0140-6736(17)32252-3 28864332

[103] Foscolou A Magriplis E Tyrovolas S Chrysohoou C Sidossis L Matalas AL. The association of protein and carbohydrate intake with successful aging: a combined analysis of two epidemiological studies. Eur J Nutr. (2019) 58:807–17. 10.1007/s00394-018-1693-2 29687264

[104] Ho FK Gray SR Welsh P Petermann-Rocha F Foster H Waddell H. Associations of fat and carbohydrate intake with cardiovascular disease and mortality: prospective cohort study of UK Biobank participants. BMJ. (2020) 368:m688. 10.1136/bmj.m688 32188587 PMC7190059

[105] Han X Ding S Lu J Li Y. Global, regional, and national burdens of common micronutrient deficiencies from 1990 to 2019: A secondary trend analysis based on the Global Burden of Disease 2019 study. EClinicalMedicine. (2022) 44:101299. 10.1016/j.eclinm.2022.101299 35198923 PMC8850322

[106] U.S. Food and Drug Administration. Health Claims; Soluble Dietary Fiber from Certain Foods and Coronary Heart Disease. (2005). Available online at: http://www.fda.gov/default.htm. (accessed 30/12/2021).

[107] EFSA Panel on Dietetic Products Nutrition and Allergies (NDA). Scientific Opinion on the substantiation of a health claim related to barley beta-glucan and lowering of blood cholesterol and reduced risk of (coronary) heart disease pursuant to Article 14 of Regulation (EC) No 1924/2006. EFSA J. (2011) 9:2470. 10.2903/j.efsa.2011.2470 PMC13093158 42016010

[108] Müller M Canfora EE Blaak EE. Gastrointestinal transit time, glucose homeostasis and metabolic health: modulation by dietary fibers. Nutrients. (2018) 10:275. 10.3390/nu10030275 29495569 PMC5872693

[109] Salamone D Rivellese AA Vetrani C. The relationship between gut microbiota, short-chain fatty acids and type 2 diabetes mellitus: the possible role of dietary fiber. Acta Diabetol. (2021) 58:1131–1138. 10.1007/s00592-021-01727-5 33970303 PMC8316221

[110] Mahendra Kumar C Singh SA. Bioactive lignans from sesame (Sesamum indicum L.): evaluation of their antioxidant and antibacterial effects for food applications. J Food Sci Technol. (2015) 52:2934–41. 10.1007/s13197-014-1334-6 25892793 PMC4397349

[111] Jeng CGK & Hou CWR. Sesamin and sesamolin: natures therapeutic lignans. Current Enzyme Inhibition. (2005) 1(1). 10.2174/1573408052952748

[112] Namiki M. Nutraceutical functions of sesame: a review. Crit Rev Food Sci Nutr. (2007) 47:651–73. 10.1080/10408390600919114 17943496

[113] Khosravi-Boroujeni H Nikbakht E Natanelov E Khalesi S. Can sesame consumption improve blood pressure? a systematic review and meta-analysis of controlled trials. J Sci Food Agric. (2017) 97:3087–94. 10.1002/jsfa.8361 28387047

[114] Yargholi A Najafi MH Zareian MA Hawkins J Shirbeigi L Ayati MH. The effects of sesame consumption on glycemic control in adults: a systematic review and meta-analysis of randomized clinical trial. Evid Based Complement Alternat Med. (2021) 2021:2873534. 10.1155/2021/2873534 34707665 PMC8545509

[115] Cofrades S López-López I Solas MT Bravo L Jiménez-Colmenero F. Influence of different types and proportions of added edible seaweeds on characteristics of low-salt gel/emulsion meat systems. Meat Sci. (2008) 79:767–76. 10.1016/j.meatsci.2007.11.010 22063041

[116] Wijesinghe WA Jeon YJ. Enzyme-assistant extraction (EAE) of bioactive components: a useful approach for recovery of industrially important metabolites from seaweeds: a review. Fitoterapia. (2012) 83:6–12. 10.1016/j.fitote.2011.10.016 22061659

[117] Cardoso SM Pereira OR Seca AM Pinto DC Silva AM. Seaweeds as preventive agents for cardiovascular diseases: from nutrients to functional foods. Mar Drugs. (2015) 13:6838–65. 10.3390/md13116838 26569268 PMC4663556

[118] Circuncisão AR Catarino MD Cardoso SM Silva AMS. Minerals from Macroalgae origin: health benefits and risks for consumers. Mar Drugs. (2018) 16:400. 10.3390/md16110400 30360515 PMC6266857

[119] Wells ML Potin P Craigie JS Raven JA Merchant SS Helliwell KE. Algae as nutritional and functional food sources: revisiting our understanding. J Appl Phycol. (2017) 29:949–82. 10.1007/s10811-016-0974-5 28458464 PMC5387034

[120] van den Driessche JJ Plat J Mensink RP. Effects of superfoods on risk factors of metabolic syndrome: a systematic review of human intervention trials. Food Funct. (2018) 9:1944–66. 10.1039/C7FO01792H 29557436

[121] EFSA Panel on Dietetic Products Nutrition and Allergies (NDA). Scientific Opinion on the substantiation of health claims related to eicosapentaenoic acid (EPA), docosahexaenoic acid (DHA), docosapentaenoic acid (DPA) and maintenance of normal cardiac function (ID 504, 506, 516, 527, 538, 703, 1128, 1317, 1324, 1325), maintenance of normal blood glucose concentrations (ID 566), maintenance of normal blood pressure (ID 506, 516, 703, 1317, 1324), maintenance of normal blood HDL-cholesterol concentrations (ID 506), maintenance of normal (fasting) blood concentrations of triglycerides (ID 506, 527, 538, 1317, 1324, 1325), maintenance of normal blood LDL-cholesterol concentrations (ID 527, 538, 1317, 1325, 4689), protection of the skin from photo-oxidative (UV-induced) damage (ID 530), improved absorption of EPA and DHA (ID 522, 523), contribution to the normal function of the immune system by decreasing the levels of eicosanoids, arachidonic acid-derived mediators and pro-inflammatory cytokines (ID 520, 2914), and “immunomodulating agent” (4690) pursuant to Article 13(1) of Regulation (EC) No 1924/2006. EFSA J. (2010) 8:1796. 10.2903/j.efsa.2010.1796

[122] Gutiérrez-Salmeán G Fabila-Castillo L Chamorro-Cevallos G. Nutritional and toxicological aspects of spirulina (ARTHROSPIRA). Nutr Hosp. (2015) 32:34–40. 10.3305/nh.2015.32.1.9001 26262693

[123] Serban MC Sahebkar A Dragan S Stoichescu-Hogea G Ursoniu S Andrica F. A systematic review and meta-analysis of the impact of Spirulina supplementation on plasma lipid concentrations. Clin Nutr. (2016) 35:842–51. 10.1016/j.clnu.2015.09.007 26433766

[124] Machowiec P Reka G Maksymowicz M Piecewicz-Szczesna H Smoleń A. Effect of spirulina supplementation on systolic and diastolic blood pressure: systematic review and meta-analysis of randomized controlled trials. Nutrients. (2021) 13:3054. 10.3390/nu13093054 34578932 PMC8468496

[125] Gabbia D De Martin S. Brown seaweeds for the management of metabolic syndrome and associated diseases. Molecules. (2020) 25:4182. 10.3390/molecules25184182 32932674 PMC7570850

[126] Izaola O Primo D Rico Bargués D Martín-Diana AB Martínez Villaluenga C Miranda J. Effects of a snack enriched with carob and Undaria pinnatifida (wakame) on metabolic parameters in a double blind, randomized clinical trial in obese patients. Nutr Hosp. (2020) 34:465–473. 10.20960/nh.02906 32379474

[127] Zaharudin N Tullin M Pekmez CT Sloth JJ Rasmussen RR Dragsted LO. Effects of brown seaweeds on postprandial glucose, insulin and appetite in humans—a randomized, 3-way, blinded, cross-over meal study. Clin Nutr. (2021) 40:830–8. 10.1016/j.clnu.2020.08.027 32917417

[128] Aakre I Solli DD Markhus MW Mæhre HK Dahl L Henjum S. Commercially available kelp and seaweed products—valuable iodine source or risk of excess intake? Food Nutr Res. (2021) 65:7584. 10.29219/fnr.v65.7584 33889064 PMC8035890

[129] Khandaker MU Chijioke NO Heffny NAB Bradley DA Alsubaie A Sulieman A. Elevated concentrations of metal(loids) in seaweed and the concomitant exposure to humans. Foods. (2021) 10:381. 10.3390/foods10020381 33578933 PMC7916668

[130] Mortensen A Kulling SE Schwartz H Rowland I Ruefer CE Rimbach G. Analytical and compositional aspects of isoflavones in food and their biological effects. Mol Nutr Food Res. (2009) 53:S266–09. 10.1002/mnfr.200800478 19774555

[131] Mueller NT Odegaard AO Gross MD Koh WP Yu MC Yuan JM. Soy intake and risk of type 2 diabetes in Chinese Singaporeans. Eur J Nutr. (2012) 51:1033–40. 10.1007/s00394-011-0290-4 22094581 PMC3480546

[132] Miller V Mente A Dehghan M Rangarajan S Zhang X Swaminathan S. Fruit, vegetable, and legume intake, and cardiovascular disease and deaths in 18 countries (PURE): a prospective cohort study. Lancet. (2017) 390:2037–49. 10.1016/S0140-6736(17)32253-5 28864331

[133] Nagata C Wada K Tamura T Konishi K Goto Y Koda S. Dietary soy and natto intake and cardiovascular disease mortality in Japanese adults: the Takayama study. Am J Clin Nutr. (2017) 105:426–31. 10.3945/ajcn.116.137281 27927636

[134] Yan Z Zhang X Li C Jiao S Dong W. Association between consumption of soy and risk of cardiovascular disease: a meta-analysis of observational studies. Eur J Prev Cardiol. (2017) 24:735–47. 10.1177/2047487316686441 28067550

[135] Konishi K Wada K Yamakawa M Goto Y Mizuta F Koda S. Dietary soy intake is inversely associated with risk of type 2 diabetes in japanese women but not in men. J Nutr. (2019) 149:1208–14. 10.1093/jn/nxz047 31079144

[136] Im J Park K. Association between soy food and dietary soy isoflavone intake and the risk of cardiovascular disease in women: a prospective cohort study in Korea. Nutrients. (2021) 13:1407. 10.3390/nu13051407 33922001 PMC8143453

[137] Nagata C. Soy intake and chronic disease risk: findings from prospective cohort studies in Japan. Eur J Clin Nutr. (2021) 75:890–901. 10.1038/s41430-020-00744-x 32917961

[138] Pirro M Vetrani C Bianchi C Mannarino MR Bernini F Rivellese AA. Joint position statement on “Nutraceuticals for the treatment of hypercholesterolemia” of the Italian Society of Diabetology (SID) and of the Italian Society for the Study of Arteriosclerosis (SISA). Nutr Metab Cardiovasc Dis. (2017) 27:2–17. 10.1016/j.numecd.2016.11.122 27956024

[139] U.S. Food and Drug Administration Health Claims. Soy protein and risk of coronary heart disease (CHD). (2014). Available online at: http://www.fda.gov/default.htm (accessed 30/12/2021).

[140] Naughton SS Mathai ML Hryciw DH McAinch AJ. Australia's nutrition transition 1961-2009: a focus on fats. Br J Nutr. (2015) 114:337–46. 10.1017/S0007114515001907 26123446

[141] Tu XH Wu BF Xie Y Xu SL Wu ZY Lv X. A comprehensive study of raw and roasted macadamia nuts: Lipid profile, physicochemical, nutritional, and sensory properties. Food Sci Nutr. (2021) 9:1688–97. 10.1002/fsn3.2143 33747479 PMC7958573

[142] Garg ML Blake RJ Wills RB Clayton EH. Macadamia nut consumption modulates favorably risk factorsfor coronary artery disease in hypercholesterolemic subjects. Lipids. (2007) 42:583–7. 10.1007/s11745-007-3042-8 17437143

[143] Griel AE Cao Y Bagshaw DD Cifelli AM Holub B Kris-Etherton PM. A macadamia nut-rich diet reduces total and LDL-cholesterol in mildly hypercholesterolemic men and women. J Nutr. (2008) 138:761–7. 10.1093/jn/138.4.761 18356332

[144] Nichols PD Glencross B Petrie JR Singh SP. Readily available sources of long-chain omega-3 oils: is farmed Australian seafood a better source of the good oil than wild-caught seafood? Nutrients. (2014) 6:1063–79. 10.3390/nu6031063 24618601 PMC3967178

[145] Lim V Gorji SG Daygon VD Fitzgerald M. Untargeted and targeted metabolomic profiling of australian indigenous fruits. Metabolites. (2020) 10:114. 10.3390/metabo10030114 32204361 PMC7143387

[146] Sacks D Baxter B Campbell BCV Carpenter JS Cognard C Dippel D. Multisociety Consensus Quality Improvement Revised Consensus Statement for Endovascular Therapy of Acute Ischemic Stroke: From the American Association of Neurological Surgeons (AANS), American Society of Neuroradiology (ASNR), Cardiovascular and Interventional Radiology Society of Europe (CIRSE), Canadian Interventional Radiology Association (CIRA), Congress of Neurological Surgeons (CNS), European Society of Minimally Invasive Neurological Therapy (ESMINT), European Society of Neuroradiology (ESNR), European Stroke Organization (ESO), Society for Cardiovascular Angiography and Interventions (SCAI), Society of Interventional Radiology (SIR), Society of NeuroInterventional Surgery (SNIS), and World Stroke Organization (WSO). J Vasc Interv Radiol. (2018) 29:441–53. 10.1016/j.jvir.2017.11.026 29478797

[147] Wang Y Ji S Zang W Wang N Cao J Li X. Identification of phenolic compounds from a unique citrus species, finger lime (Citrus australasica) and their inhibition of LPS-induced NO-releasing in BV-2 cell line. Food Chem Toxicol. (2019) 129:54–63. 10.1016/j.fct.2019.04.006 30978372

[148] Roswall N Sandin S Löf M Skeie G Olsen A Adami HO. Adherence to the healthy Nordic food index and total and cause-specific mortality among Swedish women. Eur J Epidemiol. (2015) 30:509–17. 10.1007/s10654-015-0021-x 25784368

[149] Miyagi S Iwama N Kawabata T Hasegawa K. Longevity and diet in Okinawa, Japan: the past, present and future. Asia Pac J Public Health. (2003) 15:S3–9. 10.1177/101053950301500S03 18924533

[150] Willcox DC Scapagnini G Willcox BJ. Healthy aging diets other than the Mediterranean: a focus on the Okinawan diet. Mech Aging Dev. (2014) 136:148–62. 10.1016/j.mad.2014.01.002 24462788 PMC5403516

[151] Krznarić Ž Karas I Ljubas Kelečić D Vranešić Bender D. The Mediterranean and Nordic diet: a review of differences and similarities of two sustainable, health-promoting dietary patterns. Front Nutr. (2021) 8:683678. 10.3389/fnut.2021.683678 34249991 PMC8270004

